# Integrated Trajectory Planning, MEC Offloading, and Safety Coordination for Multi-UAV Disaster Response

**DOI:** 10.3390/s26175544

**Published:** 2026-08-31

**Authors:** Rakan Armoush, Shidrokh Goudarzi, Muhammad Nadeem Khan, Alireza Esfahani

**Affiliations:** School of Computing and Engineering, University of West London, London W5 5RF, UK; 21373879@student.uwl.ac.uk (R.A.); 33126175@student.uwl.ac.uk (M.N.K.); alireza.esfahani@uwl.ac.uk (A.E.)

**Keywords:** Unmanned Aerial Vehicles (UAVs), disaster response, aerial data collection, 3D-TSPN, Age of Information (AoI), AoI-aware genetic algorithm, RRT-Connect, Lyapunov optimisation, binary MEC offloading, queue stability, 3D path planning, multi-UAV coordination, collision monitoring

## Abstract

Rapid, reliable, and energy-efficient data collection is essential for disaster response, where terrestrial communication networks may be disrupted or unavailable. Unmanned Aerial Vehicles (UAVs) provide a flexible means of collecting critical sensing data, but their operation is constrained by limited onboard energy, stochastic wireless conditions, complex three-dimensional environments, and stringent latency requirements. This paper presents a structured multi-UAV framework that separates mission optimisation into spatial, temporal, and safety layers. In the spatial layer, a three-dimensional Travelling Salesman Problem with Neighbourhoods (3D-TSPN) formulation enables UAVs to collect data by entering valid sensing regions rather than visiting exact sensor coordinates. An Age of Information (AoI)-aware Genetic Algorithm (GA) optimises the sensor-visitation sequence, while Rapidly Exploring Random Tree Connect (RRT-Connect) generates obstacle-aware feasible paths in the three-dimensional environment. In the temporal layer, a Lyapunov-based controller selects between local processing and binary offloading to a single Mobile Edge Computing (MEC) node according to queue backlog, processing delay, energy consumption, information freshness, wireless-link feasibility, and task deadlines. In the safety layer, continuous-time conflict detection and bounded temporal or spatial adjustments are used to monitor and mitigate inter-UAV and obstacle-related risks. The framework is evaluated under stochastic wireless, mobility, computation, and obstacle conditions using 20 independent random seeds. Across the corresponding 20 proposed-policy runs, it achieves a 100% mission-validity rate, complete sensor coverage, no dropped tasks, and zero final collision or near-miss events. Compared with planning-oriented and MEC-oriented baselines, the proposed framework achieves lower information age, average delay, processing delay, energy consumption, and system cost under the evaluated conditions, while maintaining reliable multi-UAV coordination. The layered design also clarifies the contribution of each component: 3D-TSPN provides spatial flexibility, the AoI-aware GA improves route sequencing, RRT-Connect supports obstacle-aware path feasibility, Lyapunov control enables queue-aware processing decisions, and safety monitoring supports coordinated multi-UAV operation. These results indicate that integrating spatial planning, computation control, and safety coordination within a clearly separated layered architecture can provide an effective solution for multi-UAV disaster-response data collection in complex three-dimensional environments.

## 1. Introduction

Unmanned Aerial Vehicles (UAVs) have become indispensable assets in modern disaster management due to their ability to rapidly deploy, hover, and navigate unsafe or inaccessible environments [1,2]. Their inherent mobility enables critical operations such as search and rescue, rapid damage assessment, real-time surveillance, and temporary communication restoration in scenarios where terrestrial infrastructure is partially or completely impaired [3,4,5]. When integrated with Internet of Things (IoT) ecosystems, UAVs act as airborne data collectors and relay platforms, connecting isolated sensor networks with emergency command and control centres  [6,7]. Prior studies have demonstrated the effectiveness of UAVs in disaster scenarios, where they often provide the only feasible sensing and communication capability when ground infrastructure fails [1,2].

Beyond direct sensing, UAVs enhance situational awareness by serving as airborne gateways in IoT-enabled disaster-response systems. They aggregate data from distributed ground sensors and relay it to command centres or edge infrastructure, thereby improving operational visibility and decision-making. In Public Safety Communications (PSC), UAVs can temporarily replace disrupted terrestrial nodes, preserving network continuity during infrastructure failures [3]. To further improve responsiveness and scalability, MEC has been explored as an enabling technology. MEC reduces latency by placing computation closer to UAVs and sensors [8,9].

Despite these advances, disaster environments impose severe and interdependent constraints on UAV operations [5,10,11]. These include complex three-dimensional (3D) obstacle fields caused by collapsed structures and debris; strict energy limitations that constrain flight endurance and sensing capabilities; stochastic wireless communication links affected by interference and infrastructure damage; and stringent latency requirements that demand fresh, timely situational information. In addition, reliable multi-UAV coordination is required to ensure safe separation and collision-free operation during concurrent missions. Addressing these challenges requires UAV systems to simultaneously plan feasible trajectories, maintain sensor coverage, collect and process data, offload computation when beneficial, and operate safely under uncertainty.

### 1.1. Related Work and Motivation

Existing research on UAV-assisted disaster response can be broadly organised around spatial path planning, sensing-neighbourhood coverage, information freshness, MEC-assisted computation, and multi-UAV coordination. Although substantial progress has been achieved in each of these directions, the corresponding components are commonly studied in isolation or connected through loosely coupled processing stages.

#### 1.1.1. Spatial Planning and Sensing-Neighbourhood Coverage

Sampling-based motion planners such as the Rapidly Exploring Random Tree (RRT), RRT-Connect, and RRT* are effective for generating collision-free paths in cluttered environments [12,13,14]. Their primary objective, however, is geometric path feasibility, and they do not generally optimise the global sensor-visitation order, information freshness, or computation-queue behaviour.

Tran et al. [15] integrated RRT* path planning with sliding-mode trajectory tracking, RBF-neural-network uncertainty compensation, and PSO-based controller tuning for robust quadrotor operation under model uncertainty and external disturbances. Their work focuses on low-level trajectory generation and tracking control, whereas the present study focuses on mission-level multi-UAV sensing, AoI-aware route sequencing, MEC processing, queue control, and inter-UAV safety.

Shi et al. [16] proposed a real-time UAV trajectory planning and tracking method that integrates deep reinforcement learning with adaptive nonlinear model predictive control. Their ANMPC-SAC framework uses a Soft Actor–Critic agent to dynamically adjust the weight matrices of the ANMPC cost function, while the receding-horizon controller generates reference trajectories and control inputs subject to nonlinear dynamics, actuator limits, and obstacle constraints. The reward model incorporates trajectory tracking, target attainment, mission completion, and trajectory smoothness. The method was evaluated in three-dimensional static and dynamic multi-obstacle environments that included moving targets, unexpected obstacles, and wind disturbances. Its main strength is real-time closed-loop adaptation and robust low-level trajectory tracking in dynamic environments. In contrast, the present work addresses mission-level coordination among multiple UAVs, including sensor assignment, sensing-neighbourhood sequencing, AoI-aware route planning, binary LOCAL/MEC processing, queue evolution, and continuous-time fleet safety. The current framework, therefore, complements, rather than replaces, the control-level DRL–ANMPC approach of Shi et al.

Metaheuristic methods, including genetic algorithms, Particle Swarm Optimisation, and other bio-inspired techniques, provide flexible global search mechanisms for UAV routing and task sequencing [17,18,19,20]. Nevertheless, these approaches often optimise distance, mission time, or energy while representing obstacle-aware execution and detailed three-dimensional motion feasibility only approximately.

Neighbourhood-based formulations, such as the Travelling Salesman Problem with Neighbourhoods (TSPN), are particularly relevant to UAV data collection because a UAV does not need to visit the exact coordinate of each sensor. Instead, data collection can occur when the UAV enters a valid sensing neighbourhood [21,22,23]. This spatial flexibility is useful in obstacle-rich disaster environments. Existing TSPN-related UAV planning methods, however, mainly emphasise trajectory length, mission completion time, or energy consumption and rarely combine sensing-neighbourhood coverage with AoI-aware route ordering, computation delay, and queue-aware processing control [23,24,25].

#### 1.1.2. AoI-Aware Sensing and MEC-Assisted Computation

Age of Information (AoI) has become an important performance metric for time-critical sensing systems because stale updates can reduce situational awareness and delay emergency response [26,27,28]. Existing AoI-aware UAV studies demonstrate the benefits of freshness-oriented scheduling and trajectory planning. However, AoI is frequently treated as a temporal scheduling objective or a post-routing performance measure rather than as a variable that directly influences obstacle-aware three-dimensional sensor sequencing [22,27,29].

MEC has also been extensively investigated as a means of reducing computation delay and relieving the limited onboard processing capacity of UAVs [8,9,30]. Existing local-versus-edge computation frameworks employ convex optimisation, mixed-integer programming, dynamic resource allocation, and Lyapunov-based control to balance latency and energy consumption. Despite these advances, many MEC formulations assume fixed, predetermined, or highly abstracted UAV mobility and do not interact directly with obstacle-aware three-dimensional path construction [30,31,32].

This separation is important in disaster missions because the spatial and temporal decisions are interdependent. The path followed by a UAV determines when each sensor is collected, the position at which communication is attempted, the UAV–MEC distance, the instantaneous wireless-link condition, and the remaining mission energy. These quantities subsequently affect the feasibility and cost of local processing or MEC offloading, the task-queue evolution, and the completion-time AoI of the collected data.

#### 1.1.3. Multi-UAV Coordination and Layered Optimisation

Concurrent multi-UAV missions introduce additional challenges, including overlapping trajectories, asynchronous task completion, and inter-UAV collision risk [33,34,35]. Existing coordination methods include rule-based deconfliction, Multi-Agent Path Finding, Conflict-Based Search, Safe Interval Path Planning, and time-expanded planning formulations. Although some of these methods provide formal safety guarantees, they often rely on discretised spatial or temporal representations whose assumptions may limit direct application to continuous three-dimensional disaster-response environments [33,34,35].

Layered approaches that separate global route optimisation from local motion planning provide a practical alternative. Such architectures improve modularity and computational tractability by assigning different responsibilities to task allocation, route sequencing, geometric path generation, computation control, and safety monitoring. However, existing designs commonly treat spatial planning, AoI management, MEC offloading, queue evolution, and conflict resolution as separate or sequential processes with limited exchange of system state.

Table 1 provides a paper-specific comparison of representative spatial-planning, AoI-aware sensing, MEC-assisted computation, and multi-agent coordination studies. The comparison identifies the problem addressed, the adopted method, the principal strength, and the remaining limitation of each study relative to the proposed framework. The reviewed works provide strong solutions within their respective domains, including robust trajectory tracking, energy-aware data collection, AoI minimisation, computation offloading, server deployment, and collision-free planning. However, none of the representative studies jointly connects sensing-neighbourhood coverage, AoI-aware route sequencing, obstacle-aware three-dimensional path construction, queue-aware LOCAL/MEC processing, and continuous-time multi-UAV safety assessment within one layered mission architecture. Carrese et al. [36] studied the coordinated use of UAVs and human beautificators for managing improperly parked e-scooters. Their objective is to maximise the total monetary value of beautification, seeking, movement, waiting, and battery-swapping actions over a discretised time horizon. The problem is formulated as a mixed-integer linear program on a time–space network, with an unsplittable multicommodity-flow structure representing the actions of UAVs and beautificators. To accelerate CPLEX, the authors proposed a warm-start heuristic based on partial removal of movement arcs, followed by a RINS-based ILP improvement procedure. The main strength of this study is its rigorous operational scheduling model, which captures UAV battery limits, battery swapping, human–UAV interaction, and action consistency over space and time. However, its scope is e-scooter fleet beautification and urban-decorum management rather than multi-UAV disaster-response sensing. It does not address sensing-neighbourhood coverage, AoI-aware route sequencing, obstacle-aware continuous 3D trajectory construction, LOCAL/MEC processing, computation queues, or continuous-time inter-UAV safety.

### 1.2. Research Gap and Proposed Direction

The literature, therefore, reveals an integration gap rather than an absence of individual planning or control techniques. Existing AoI-aware UAV-routing methods seldom incorporate obstacle-aware three-dimensional path realisation, task-queue evolution, and local/MEC processing decisions within the same framework. Conversely, MEC-offloading studies commonly abstract UAV mobility or assume fixed trajectories and, therefore, do not directly consider sensing-neighbourhood coverage, obstacle-aware route execution, or continuous multi-UAV deconfliction. Spatial planning, information freshness, computation control, and safety coordination are, consequently, often optimised separately, despite their direct interdependence in disaster-response missions.

To address this gap, the proposed framework is organised into three interacting layers. The spatial layer combines three-dimensional Travelling Salesman Problem with Neighbourhoods (3D-TSPN) modelling, AoI-aware genetic algorithm (GA) sensor sequencing, and RRT-Connect-based path construction. The temporal layer applies Lyapunov drift-plus-penalty control to select between local processing and binary MEC offloading according to queue backlog, processing delay, energy consumption, completion-time AoI, wireless-link feasibility, and task deadlines. The safety layer monitors inflated-obstacle clearance and continuous-time inter-UAV separation and applies bounded temporal holds or altitude adjustments when required.

The principal novelty lies in the structured interfaces among these layers. Spatial collection events determine task arrivals, UAV positions, and communication conditions for the temporal controller, while the safety layer adjusts mission timing or altitude without changing the required sensing coverage. The contribution is, therefore, the coordinated integration of established planning, computation-control, and safety methods rather than a claim that 3D-TSPN, GA, RRT-Connect, Lyapunov optimisation, or MEC offloading is independently new.

The main contributions of this work are summarised as follows:

(1) A layered multi-UAV disaster-response architecture is developed to connect spatial sensing and path-planning decisions with temporal computation control and continuous-time safety assessment through shared mission states.
(2) A spatial planning layer combines 3D-TSPN sensing neighbourhoods, AoI-aware GA sensor sequencing, and RRT-Connect path construction under sensing-coverage and inflated-obstacle constraints.
(3) A Lyapunov-based temporal layer selects between local processing and binary MEC offloading according to queue backlog, processing delay, energy consumption, completion-time AoI, wireless-link feasibility, and task deadlines.
(4) A hybrid safety mechanism evaluates continuous-time closest approach and applies bounded temporal holds or altitude adjustments while preserving obstacle clearance and sensing coverage.
(5) The complete framework is evaluated over 20 independent stochastic simulation runs and against planning-oriented and computation-oriented baselines using coverage, information freshness, delay, energy, queue, fairness, computational, and safety metrics.

Together, these contributions define a layered spatial–temporal architecture that integrates sensing, path planning, computation control, and safety assessment under the evaluated disaster-response conditions.

The remainder of this paper is organised as follows. Section 2 presents the system model, including UAV mobility, quadrotor dynamics, sensing-neighbourhood coverage, communication, binary MEC offloading, queue evolution, AoI, energy, and safety constraints. Section 3 develops the Lyapunov-based temporal control model and its conditional queue-stability and cost-optimality analysis. Section 4 describes the proposed layered multi-UAV framework, including 3D-TSPN modelling, AoI-aware GA sequencing, RRT-Connect path construction, adaptive path recovery, and multi-UAV deconfliction. Section 5 presents the simulation setup, while Section 6 reports the experimental results, robustness analysis, ablation studies, and benchmark comparisons. Finally, Section 7 concludes the paper and outlines future research directions.

## 2. System Model

This section provides the mathematical formalisation of the UAV-assisted disaster-response system. The model captures UAV mobility, sensing-neighbourhood coverage, data generation, local and MEC-assisted task processing, wireless communication, queue evolution, AoI dynamics, and energy consumption. The subsequent optimisation modules, including AoI-aware GA sequencing, RRT-Connect path feasibility checking, and Lyapunov-based binary MEC offloading, operate on this model.

We consider a three-dimensional disaster region of dimensions $L_{x}\times L_{y}\times H$, which contains *N* static ground sensors, *U* concurrently deployed UAVs, a set of obstacles O, and a single edge node located at position e. The set of UAVs is denoted by U={1,…,U}, and the set of sensors is denoted by S={1,…,N}.

Throughout the manuscript, i∈U indexes UAVs, j∈S indexes sensors and their associated computation tasks, *t* denotes the decision slot, and m∈{loc,mec} denotes the processing mode. Task-specific quantities carry the index *j*, while quantities related to the collecting UAV additionally carry the index *i*. The scalar $Q_{i}\left(t\right)$ denotes the queue backlog of UAV *i*, whereas Q(t) denotes the aggregate queue-state vector.

UAVs are required to collect data from all assigned sensors, maintain safe navigation in a 3D-obstacle-rich environment, and decide whether each collected task should be processed locally or offloaded to the edge node.

### 2.1. UAV Mobility Model

Each UAV i∈U operates under the following discrete-time kinematic model:

(1) $$\begin{array}{c} p_{i}\left(t+1\right)=p_{i}\left(t\right)+v_{i}\left(t\right)\Delta t, \end{array}$$

where $p_{i}\left(t\right)\in R^{3}$ and $v_{i}\left(t\right)\in R^{3}$ denote the position and velocity of UAV *i* at time *t*, respectively. The UAV velocity is constrained by

(2) $$\begin{array}{c} ∥v_{i}\left(t\right)∥\leq v_{\max}, \end{array}$$

where $v_{\max}$ is the maximum allowable UAV speed [4,37].

For obstacle and safety modelling, each obstacle $O_{k}\in O$ is inflated by a safety margin $d_{\mathrm{safe}}$:

(3) $$\begin{array}{c} O_{k}^{+}=O_{k}⊕B\left(d_{\mathrm{safe}}\right), \end{array}$$

where $B(d_{\mathrm{safe}})$ denotes a ball of radius $d_{\mathrm{safe}}$. This inflation provides a conservative representation of obstacles and supports robust collision avoidance under modelling and localisation uncertainty.

The discrete-time model in (1) is used by the mission-level planner to represent the translational reference motion of each UAV over the planning horizon. However, a physical quadrotor also exhibits coupled translational and rotational dynamics. Therefore, the following subsection introduces the underlying quadrotor model and clarifies the relationship between the generated kinematic reference trajectory and the low-level flight-control system.

### 2.2. Quadrotor Dynamic Model and Planning Abstraction

The discrete-time mobility model introduced above represents the mission-level translational motion used by the proposed spatial planner. To clarify the physical interpretation of the aerial platform, each UAV is additionally regarded as a rigid-body quadrotor with coupled translational and rotational dynamics.

Let $p_{i}={\left[x_{i},y_{i},z_{i}\right]}^{T}\in R^{3}$ and $v_{i}={\dot{p}}_{i}$ denote the inertial-frame position and velocity of UAV *i*, respectively. Its attitude is represented by

(4) $$\eta_{i}={\left[\begin{array}{ccc} \phi_{i} & \theta_{i} & \psi_{i} \end{array}\right]}^{T},$$

where $\phi_{i}$, $\theta_{i}$, and $\psi_{i}$ are the roll, pitch, and yaw angles. The body-frame angular velocity is denoted by

(5) $$\omega_{i}={\left[\begin{array}{ccc} p_{i}^{\omega } & q_{i}^{\omega } & r_{i}^{\omega } \end{array}\right]}^{T}.$$

The translational rigid-body dynamics can be expressed as

(6) $$m_{i}{\ddot{p}}_{i}=m_{i}g+R_{i}\left(\phi_{i},\theta_{i},\psi_{i}\right)\left[\begin{array}{c} 0\\ 0\\ f_{T,i} \end{array}\right]-f_{D,i},$$

where $m_{i}$ is the UAV mass, g is the gravitational acceleration vector, $R_{i}\in SO\left(3\right)$ is the body-to-inertial rotation matrix, $f_{T,i}$ is the collective rotor thrust, and $f_{D,i}$ represents aerodynamic drag and unmodelled translational disturbances. The collective thrust is

(7) $$f_{T,i}=\sum_{k=1}^{4}F_{i,k},$$

where $F_{i,k}$ is the thrust generated by rotor *k*.

The rotational dynamics are described by

(8) $$J_{i}{\dot{\omega }}_{i}+\omega_{i}\times \left(J_{i}\omega_{i}\right)=\tau_{i},$$

where $J_{i}$ is the inertia matrix and $\tau_{i}={\left[\tau_{\phi ,i},\tau_{\theta ,i},\tau_{\psi ,i}\right]}^{T}$ contains the roll, pitch, and yaw control torques. The corresponding attitude kinematics are

(9) $${\dot{R}}_{i}=R_{i}{\left[\omega_{i}\right]}_{\times },$$

where ${\left[\omega_{i}\right]}_{\times }$ is the skew-symmetric matrix associated with $\omega_{i}$.

**Figure 1:**
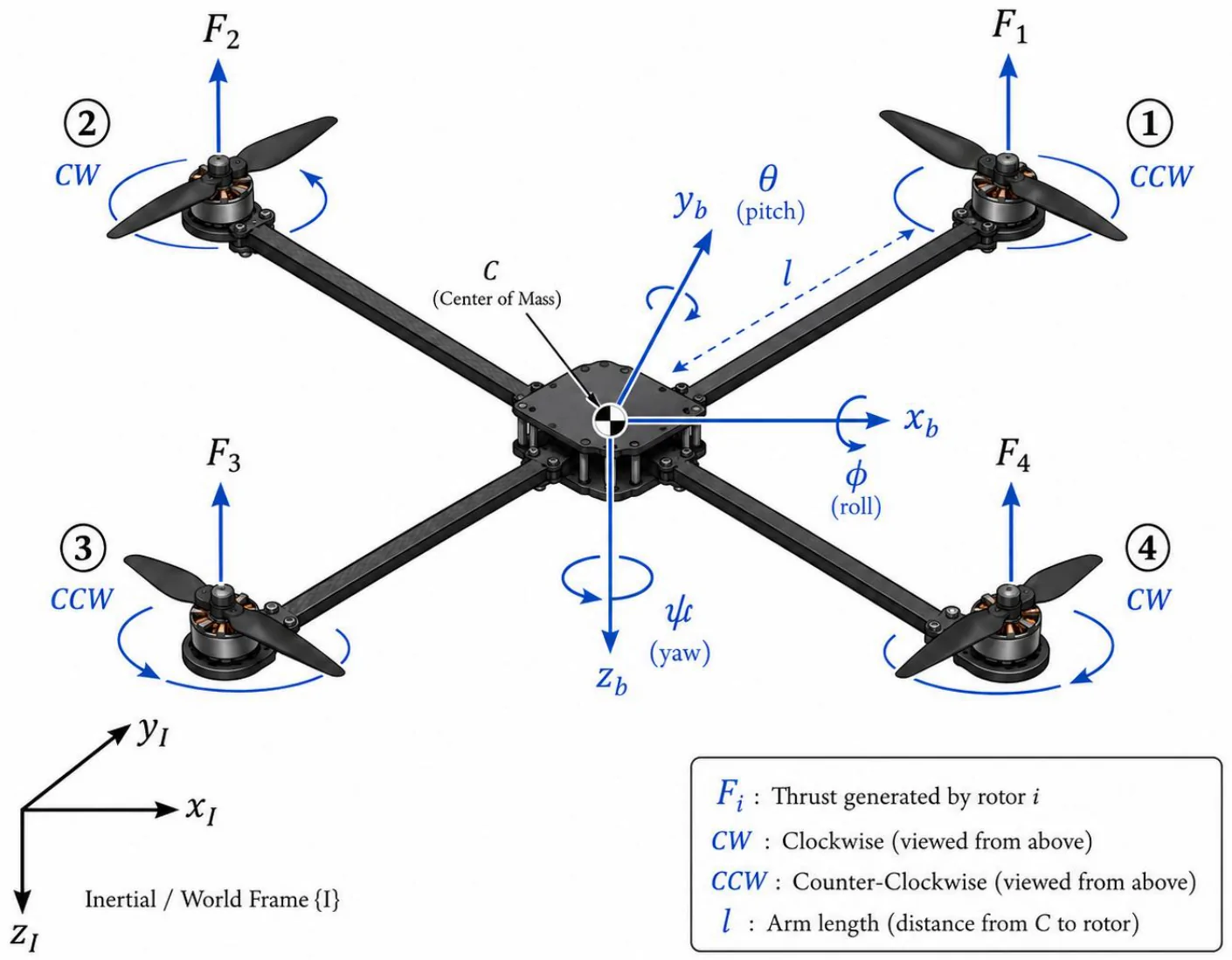
Quadrotor configuration showing the body-fixed axes, individual rotor thrusts, and roll, pitch, and yaw motions.

For the quadrotor configuration shown in Figure 1, the four rotor thrusts generate the collective thrust and body torques. A generic actuator mapping is

(10) $$\left[\begin{array}{c} f_{T,i}\\ \tau_{\phi ,i}\\ \tau_{\theta ,i}\\ \tau_{\psi ,i} \end{array}\right]=\left[\begin{array}{cccc} 1 & 1 & 1 & 1\\ 0 & l_{i} & 0 & -l_{i}\\ -l_{i} & 0 & l_{i} & 0\\ \kappa_{i} & -\kappa_{i} & \kappa_{i} & -\kappa_{i} \end{array}\right]\left[\begin{array}{c} F_{i,1}\\ F_{i,2}\\ F_{i,3}\\ F_{i,4} \end{array}\right],$$

where $l_{i}$ is the arm length and $\kappa_{i}$ is the rotor drag-to-thrust coefficient.

The proposed optimisation framework operates at the mission-planning level rather than at the motor-control level. In the implemented simulation, the 3D-TSPN–GA–RRT-Connect module generates a sequence of collision-free position waypoints. The path is then spatially resampled, and its mission timeline is constructed using the prescribed constant reference speed $v_{\mathrm{uav}}=1$ m/s. Therefore, the generated path is interpreted as a position-reference trajectory,

(11) $$p_{i}^{\mathrm{ref}}\left(t\right),$$

to be tracked by a lower-level position and attitude controller.

The current implementation explicitly validates map boundaries, sensing-neighbourhood coverage, inflated-obstacle avoidance, and continuous-time inter-UAV separation. It does not explicitly simulate motor speeds, rotor saturation, attitude transients, or angular-rate constraints. Accordingly, the rigid-body equations above provide the physical quadrotor interpretation of the mission-level reference trajectory, while detailed flight-controller design and motor-level dynamic validation remain outside the scope of the present study.

### 2.3. Sensing and Coverage Model

Each ground sensor $s_{j}\in S$ is located at position $s_{j}\in R^{3}$. Instead of requiring the UAV to visit the exact sensor coordinate, each sensor is associated with a sensing neighbourhood:

(12) $$\begin{array}{c} N_{j}=\left\{x\in R^{3}:∥x-s_{j}∥\leq R_{s}\right\}, \end{array}$$

where $R_{s}$ is the sensing radius.

A sensor *j* is considered successfully collected by UAV *i* if

(13) $$\begin{array}{c} p_{i}\left(t\right)\in N_{j}. \end{array}$$

This transforms the coverage problem into a three-dimensional Travelling Salesman Problem with Neighbourhoods (3D-TSPN), where the UAV must visit sensing regions rather than exact sensor coordinates. This formulation increases spatial flexibility and allows the planner to select feasible collection points that avoid obstacles while still satisfying sensing requirements [21,22,23].

In the considered disaster-response mission, each sensor generates one data update, which is collected at most once. A sensor is marked as collected when its assigned UAV first enters the corresponding sensing neighbourhood. After this first successful collection, the sensor is removed from the set of unvisited sensing targets and is not revisited during the same mission.

### 2.4. Task Processing and Binary MEC-Offloading Model

Each sensor *j* generates a data task of size $d_{j}$ bits with a processing deadline $\tau_{j}$. Only one task is generated by each sensor during the considered mission. Once the task is collected by a UAV, it can be processed in one of two modes:

(14) $$\begin{array}{c} x_{ij}\left(t\right)\in \left\{0,1\right\}, \end{array}$$

where

(15) $$\begin{array}{c} x_{ij}\left(t\right)=\left\{\begin{array}{cc} 0, & \mathrm{local}\;\mathrm{processing}\;\mathrm{at}\;\mathrm{the}\;\mathrm{UAV},\\ 1, & \mathrm{full}\;\mathrm{offloading}\;\mathrm{to}\;\mathrm{the}\;\mathrm{edge}\;\mathrm{node}. \end{array}\right. \end{array}$$

Thus, the computation model is binary: each task is either processed locally or fully offloaded to the edge node. Partial offloading is not considered in this framework. Each computation task is assumed to be indivisible and must be processed entirely in one mode. The onboard UAV CPU capacity is fixed during mission execution, and dynamic CPU-frequency scaling is not considered. The current binary model also does not support parallel local and MEC processing of different portions of the same task. These assumptions simplify the task-level feasibility analysis and improve the interpretability of the Lyapunov decision, but they may limit performance for divisible or computation-intensive workloads.

#### 2.4.1. Local Processing

For local processing, the required number of CPU cycles for task *j* is

(16) $$\begin{array}{c} C_{j}=d_{j}c_{j}, \end{array}$$

where $c_{j}$ is the number of CPU cycles required per bit. If UAV *i* has local computational capacity $f_{i}^{\mathrm{uav}}$, the local processing delay is

(17) $$\begin{array}{c} T_{ij}^{\mathrm{loc}}=\frac{C_{j}}{f_{i}^{\mathrm{uav}}}. \end{array}$$

The corresponding local computation energy is modelled as

(18) $$\begin{array}{c} E_{ij}^{\mathrm{loc}}=P_{i}^{\mathrm{cpu}}T_{ij}^{\mathrm{loc}}, \end{array}$$

where $P_{i}^{\mathrm{cpu}}$ denotes the computation power of UAV *i*.

#### 2.4.2. MEC Offloading

When a task is offloaded to the edge node, the uplink transmission rate from UAV *i* to the edge node is expressed as

(19) $$\begin{array}{c} R_{i}\left(t\right)=B_{i}\left(t\right)\log_{2}\left(1+\frac{P_{i}\left(t\right)h_{i}\left(t\right)}{\sigma_{i}^{2}\left(t\right)+I_{i}\left(t\right)}\right), \end{array}$$

where $B_{i}\left(t\right)$ is the available bandwidth, $P_{i}\left(t\right)$ is the transmission power, $h_{i}\left(t\right)$ is the channel gain, $\sigma_{i}^{2}\left(t\right)$ is the noise power, and $I_{i}\left(t\right)$ is the interference level [4,5,11].

The transmission delay for task *j* is

(20) $$\begin{array}{c} T_{ij}^{\mathrm{tx}}\left(t\right)=\frac{d_{j}}{R_{i}\left(t\right)}. \end{array}$$

The MEC-side processing delay is

(21) $$\begin{array}{c} T_{j}^{\mathrm{edge}}=\frac{C_{j}}{f^{\mathrm{mec}}}, \end{array}$$

where $f^{\mathrm{mec}}$ denotes the CPU capacity of the MEC node.

Therefore, the total MEC-assisted processing delay is

(22) $$\begin{array}{c} T_{ij}^{\mathrm{mec}}\left(t\right)=T_{ij}^{\mathrm{tx}}\left(t\right)+T_{j}^{\mathrm{edge}}. \end{array}$$

The communication energy consumed during offloading is

(23) $$\begin{array}{c} E_{ij}^{\mathrm{tx}}\left(t\right)=P_{i}\left(t\right)T_{ij}^{\mathrm{tx}}\left(t\right). \end{array}$$

A task is considered feasible under mode $x_{ij}\left(t\right)$ only if its delay and energy requirements can be satisfied within the current UAV battery and deadline constraints.

### 2.5. Queueing and Task-Arrival Model

Each UAV maintains a task queue $Q_{i}\left(t\right)$, which represents the accumulated computation workload awaiting service. The task-arrival process $A_{i}\left(t\right)$ is determined by the first successful collection times of the sensors along the planned UAV trajectories, and the stochastic communication conditions.

The queue evolves according to

(24) $$\begin{array}{c} Q_{i}\left(t+1\right)=\max\left[Q_{i}\left(t\right)-\mu_{i}\left(t\right),0\right]+A_{i}\left(t\right), \end{array}$$

where $\mu_{i}\left(t\right)$ is the service rate achieved by the selected processing mode. Since the offloading decision is binary, $\mu_{i}\left(t\right)$ depends on whether the task is processed locally or offloaded to the MEC node:

(25) $$\begin{array}{c} \mu_{i}\left(t\right)=\left\{\begin{array}{cc} \mu_{i}^{\mathrm{loc}}\left(t\right), & x_{ij}\left(t\right)=0,\\ \mu_{i}^{\mathrm{mec}}\left(t\right), & x_{ij}\left(t\right)=1. \end{array}\right. \end{array}$$

The stochastic queue is stabilised by the Lyapunov-based temporal controller described in the following sections.

### 2.6. Age of Information Model

Age of Information (AoI) is used to quantify the elapsed time between the generation or availability of a sensor update and the time at which that update reaches a specified mission stage. Let $g_{j}$ denote the generation or availability time of the single update associated with sensor *j*. In the present simulation, all sensor updates are assumed to be available at the beginning of the mission; hence,

(26) $$g_{j}=0,\;\forall j\in S.$$

Let $t_{ij}^{\mathrm{col}}$ denote the first time at which UAV *i* enters the sensing neighbourhood of sensor *j* and successfully collects its update. The collection-level AoI is defined as

(27) $$\Delta_{ij}^{\mathrm{col}}=t_{ij}^{\mathrm{col}}-g_{j}.$$

The collection event does not generate a new update and, therefore, does not reset the AoI to zero. Instead, it terminates the spatial waiting period and initiates the selected local- or MEC-processing operation.

Let $T_{ij}^{m}\left(t\right)$ denote the post-collection processing delay for task *j*, collected by UAV *i*, under processing mode m∈{loc,mec}. The corresponding completion-time AoI is

(28) $$\Delta_{ij}^{\mathrm{comp},m}\left(t\right)=t_{ij}^{\mathrm{col}}-g_{j}+T_{ij}^{m}\left(t\right).$$

Therefore, the collection-level AoI measures the freshness effect of sensor assignment, route sequencing, and spatial execution, whereas the completion-time AoI additionally captures the delay introduced by local processing or MEC offloading. Mission completion time is reported as a separate system-level metric and is not used as a substitute for per-update AoI.

### 2.7. Energy-Consumption Model

The overall energy consumption of each UAV consists of flight energy, communication energy, and onboard computation energy. The resulting energy quantities should be interpreted as normalised mission-energy measures used for comparative evaluation rather than as hardware-level propulsion estimates for a particular quadrotor platform.

The total energy consumption of UAV *i* is modelled as

(29) $$\begin{array}{c} E_{i}=E_{i}^{\mathrm{fly}}+E_{i}^{\mathrm{tx}}+E_{i}^{\mathrm{cpu}}, \end{array}$$

where $E_{i}^{\mathrm{fly}}$, $E_{i}^{\mathrm{tx}}$, and $E_{i}^{\mathrm{cpu}}$ denote flight, communication, and local computation energy components, respectively.

The flight energy is related to the mission flight time and UAV propulsion power:

(30) $$\begin{array}{c} E_{i}^{\mathrm{fly}}=P_{i}^{\mathrm{fly}}T_{i}^{\mathrm{fly}}, \end{array}$$

where $P_{i}^{\mathrm{fly}}$ denotes the UAV flight or hovering power and $T_{i}^{\mathrm{fly}}$ is the total flight time.

The communication energy consumed for offloading task *j* is

(31) $$\begin{array}{c} E_{ij}^{\mathrm{tx}}\left(t\right)=P_{i}\left(t\right)T_{ij}^{\mathrm{tx}}\left(t\right). \end{array}$$

The onboard computation energy for local processing is

(32) $$\begin{array}{c} E_{ij}^{\mathrm{cpu}}=P_{i}^{\mathrm{cpu}}T_{ij}^{\mathrm{loc}}. \end{array}$$

The UAV battery constraint is expressed as

(33) $$\begin{array}{c} E_{i}\leq E_{i}^{\max}, \end{array}$$

where $E_{i}^{\max}$ denotes the initial energy capacity of UAV *i*.

### 2.8. End-to-End Delay Model

The end-to-end delay of a collected task consists of the processing delay and, if offloaded, the transmission delay. For task *j* collected by UAV *i*, the total delay is modelled as

(34) $$\begin{array}{c} T_{ij}\left(t\right)=\left\{\begin{array}{cc} T_{ij}^{\mathrm{loc}}\left(t\right),\; & x_{ij}\left(t\right)=0,\\ T_{ij}^{\mathrm{mec}}\left(t\right),\; & x_{ij}\left(t\right)=1. \end{array}\right. \end{array}$$

The task is feasible if

(35) $$\begin{array}{c} T_{ij}\left(t\right)\leq \tau_{j}. \end{array}$$

This delay model is incorporated into the Lyapunov drift-plus-penalty framework to jointly minimise latency-related cost and maintain queue stability.

### 2.9. Safety and Collision Constraints

For safe multi-UAV operation, the minimum separation between any two UAVs must satisfy

(36) $$\begin{array}{c} ∥p_{i}\left(t\right)-p_{k}\left(t\right)∥\geq d_{\min},\;\forall i\neq k, \end{array}$$

where $d_{\min}$ is the required inter-UAV safety distance.

Obstacle avoidance is enforced by ensuring that UAV positions and path segments do not intersect the inflated obstacle set:

(37) $$\begin{array}{c} p_{i}\left(t\right)\notin O_{k}^{+},\;\forall O_{k}\in O. \end{array}$$

These constraints are monitored during mission execution using continuous-time conflict detection and safety-buffer checking.

### 2.10. Overall Optimisation Problem and Layered Decomposition

The overall mission problem couples sensor assignment, sensing-neighbourhood sequencing, three-dimensional path construction, binary task processing, queue evolution, and multi-UAV safety. The relevant index sets are the UAV set U={1,…,U}, the sensor and task set S={1,…,N}, the obstacle set O, and the processing-mode set

M={loc,mec}.

The principal parameters include the sensor position $s_{j}$, sensing radius $R_{s}$, task size $d_{j}$, required CPU cycles per bit $c_{j}$, task deadline $\tau_{j}$, UAV and MEC CPU capacities $f_{i}^{\mathrm{uav}}$ and $f^{\mathrm{mec}}$, maximum UAV speed $v_{\max}$, residual UAV energy $E_{i}^{\mathrm{rem}}\left(t\right)$, inflated obstacle regions $O_{k}^{+}$, and minimum inter-UAV separation $d_{\min}$.

The main decision variables are defined as follows:

$$a_{ij}\in \left\{0,1\right\},$$

where $a_{ij}=1$ if sensor *j* is assigned to UAV *i*; $\pi_{i}$, which denotes the ordered sequence of sensing neighbourhoods visited by UAV *i*; $q_{ij}\in R^{3}$, which denotes the selected collection point for sensor *j*; and $p_{i}\left(t\right)\in R^{3}$, which denotes the resulting UAV trajectory. The binary processing variable is

$$x_{ij}\left(t\right)\in \left\{0,1\right\},$$

where $x_{ij}\left(t\right)=0$ denotes LOCAL processing and $x_{ij}\left(t\right)=1$ denotes full MEC offloading. The safety layer may additionally apply bounded waiting, speed, or altitude corrections, denoted collectively by $\zeta_{i}\left(t\right)$.

The consolidated feasibility conditions are

(38) $$\begin{array}{cccc} \sum_{i\in U}a_{ij} & =1,\; & \; & \forall j\in S, \end{array}$$

(39) $$\begin{array}{cccc} ∥q_{ij}-s_{j}{∥}_{2} & \leq R_{s},\; & \; & \forall \left(i,j\right):a_{ij}=1, \end{array}$$

(40) $$\begin{array}{cccc} p_{i}\left(t\right) & \in A,\; & \; & \forall i,t, \end{array}$$

(41) $$\begin{array}{cccc} p_{i}\left(t\right) & \notin O_{k}^{+},\; & \; & \forall i,t,\;k\in O, \end{array}$$

(42) $$\begin{array}{cccc} p_{i}\left(t+1\right) & =p_{i}\left(t\right)+v_{i}\left(t\right)\Delta t,\; & \; & \forall i,t, \end{array}$$

(43) $$\begin{array}{cccc} ∥v_{i}{\left(t\right)∥}_{2} & \leq v_{\max},\; & \; & \forall i,t, \end{array}$$

(44) $$\begin{array}{cccc} T_{ij}^{m}\left(t\right) & \leq \tau_{j},\; & \; & \forall m\in M_{ij}\left(t\right), \end{array}$$

(45) $$\begin{array}{cccc} E_{ij}^{m}\left(t\right) & \leq E_{i}^{\mathrm{rem}}\left(t\right),\; & \; & \forall m\in M_{ij}\left(t\right), \end{array}$$

(46) $$\begin{array}{cccc} Q_{i}\left(t+1\right) & =\max\left[Q_{i}\left(t\right)-\mu_{i}\left(t\right),0\right]+A_{i}\left(t\right),\; & \; & \forall i,t, \end{array}$$

(47) $$\begin{array}{cccc} ∥p_{i}\left(t\right)-p_{k}{\left(t\right)∥}_{2} & \geq d_{\min},\; & \; & \forall i\neq k,\;t. \end{array}$$

For MEC processing, membership in the feasible-mode set $M_{ij}\left(t\right)$ additionally requires an available wireless link, $R_{i}\left(t\right)>0$, and satisfaction of the transmission, edge-processing, deadline, and residual-energy conditions defined in Section 3.

The complete problem is not solved as a single monolithic mathematical program. Instead, it is addressed through three coordinated subproblems.

First, the spatial layer determines $\left\{a_{ij},\pi_{i},q_{ij},p_{i}\left(t\right)\right\}$ by minimising the implemented route-fitness function

(48) $$\min J_{\mathrm{sp}}=w_{d}T_{\mathrm{fly}}+w_{a}\Delta_{\mathrm{AoI}}+w_{s}S_{\mathrm{smooth}},$$

subject to the assignment, sensing-coverage, map-boundary, obstacle, path, and mobility constraints in (38)–(43).

Second, after each collection event, the temporal layer selects the feasible LOCAL or MEC mode according to

(49) $$m_{ij}^{★}\left(t\right)=\arg\min_{m\in M_{ij}\left(t\right)}\left[D_{i}^{m}\left(t\right)+Vg_{ij}^{m}\left(t\right)\right],$$

where $D_{i}^{m}\left(t\right)$ is the candidate one-step queue-drift term and $g_{ij}^{m}\left(t\right)$ combines energy consumption, processing delay, and completion-time AoI.

Third, the safety layer evaluates the complete mission timeline and applies a bounded correction $\zeta_{i}\left(t\right)$ when the separation constraint in (47) is violated. Its primary objective is to eliminate unsafe UAV-pair events,

(50) $$\min_{\left\{\zeta_{i}\right\}}\sum_{i<k}I\left\{d_{ik}^{\min}<d_{\min}\right\},$$

while preserving sensing coverage, obstacle clearance, map feasibility, and the validated trajectory endpoints. Among feasible corrective actions, the implementation favours bounded changes that limit additional delay and trajectory modification.

Accordingly, the framework uses a hierarchical decomposition rather than an artificial global objective. The spatial layer first generates validated coverage-preserving trajectories and collection events. The safety layer then constructs and audits the fleet mission timelines and applies bounded temporal or spatial corrections when required. Finally, the temporal layer evaluates the LOCAL and MEC candidates at each collection event using the current queue, energy, wireless, delay, and AoI states. The resulting trajectories, processing decisions, queue states, and safety outcomes are subsequently subjected to the final mission-validity audit.

## 3. Lyapunov-Based Optimisation Framework

This section develops the Lyapunov-based temporal optimisation model for UAV task execution and binary MEC offloading. The objective is to stabilise each UAV task queue while selecting, for each collected task, whether it should be processed locally or fully offloaded to a single MEC node. The temporal layer, therefore, complements the spatial planning layer by controlling computation and communication decisions after data collection events occur.

Lyapunov optimisation is particularly suitable for disaster-response environments, where wireless channel quality, task arrivals, UAV positions, and available energy are stochastic. In the proposed framework, the Lyapunov controller does not optimise the UAV route directly. Instead, it receives collection events from the spatial layer and makes queue-aware local/MEC processing decisions based on delay, energy consumption, AoI, and queue backlog.

### 3.1. Queue Evolution

Each UAV i∈U maintains a task queue $Q_{i}\left(t\right)$, representing the accumulated workload awaiting processing. The queue evolves as

(51) $$\begin{array}{c} Q_{i}\left(t+1\right)=\max\left[Q_{i}\left(t\right)-\mu_{i}\left(t\right),0\right]+A_{i}\left(t\right), \end{array}$$

where $A_{i}\left(t\right)$ is the task-arrival process induced by sensor collection events, and $\mu_{i}\left(t\right)$ is the service rate achieved by the selected processing mode.

Since the proposed computation model is binary, each task is either processed locally or fully offloaded to the MEC node. Therefore, the service rate is defined as

(52) $$\begin{array}{c} \mu_{i}\left(t\right)=\left\{\begin{array}{cc} \mu_{i}^{\mathrm{loc}}\left(t\right), & x_{ij}\left(t\right)=0,\\ \mu_{i}^{\mathrm{mec}}\left(t\right), & x_{ij}\left(t\right)=1, \end{array}\right. \end{array}$$

where $x_{ij}\left(t\right)\in \left\{0,1\right\}$, $x_{ij}\left(t\right)=0$ denotes local processing, and $x_{ij}\left(t\right)=1$ denotes MEC offloading.

### 3.2. Boundedness and Feasibility Assumptions

The Lyapunov analysis is established under the following standard assumptions.

**Assumption 1**  (Bounded arrivals and service)**.** *For every UAV i∈U and every decision slot t, the task arrival and service processes satisfy*

(53) $$0\leq A_{i}\left(t\right)\leq A_{i}^{\max},\;0\leq \mu_{i}\left(t\right)\leq \mu_{i}^{\max},$$

*where $A_{i}^{\max}<\infty$ and $\mu_{i}^{\max}<\infty$ are finite constants.*

**Assumption 2**  (Bounded instantaneous cost)**.** *The aggregate instantaneous penalty*

(54) $$g\left(t\right)=\sum_{i=1}^{U}g_{i}\left(t\right)$$

*is uniformly bounded as*

(55) $$g_{\min}\leq g\left(t\right)\leq g_{\max},$$

*where $g_{\min}$ and $g_{\max}$ are finite constants. This follows from the finite task-size, deadline, transmission-power, computation-power, and battery constraints imposed by the system model.*

**Assumption 3**  (Existence of a stabilising policy)**.** *The mean arrival-rate vector lies strictly inside the network stability region. Hence, there exists a stationary randomised policy $\pi^{\epsilon }$ and a constant ϵ>0 such that, for every UAV,*

(56) $$E\left[\mu_{i}^{\pi^{\epsilon }}\left(t\right)-A_{i}\left(t\right)\right]\geq \epsilon .$$

*Let $g^{\epsilon }$ denote the time-average cost achieved by this ϵ-slack stationary policy.*

**Assumption 4**  (Existence of a stationary cost-optimal policy)**.** *There exists a stationary randomised policy $\pi^{★}$ that attains the optimal feasible time-average cost $g^{★}$ and satisfies*

(57) $$E\left[A_{i}\left(t\right)-\mu_{i}^{\pi^{★}}\left(t\right)\right]\leq 0,\;\forall i\in U.$$

*These assumptions separate the analytical infinite-horizon stability result from the finite-duration queue behaviour observed in the simulations.*

### 3.3. Lyapunov Function and Drift

To analyse queue stability, we define the quadratic Lyapunov function as

(58) $$\begin{array}{c} L\left(Q\left(t\right)\right)=\frac{1}{2}\sum_{i=1}^{U}Q_{i}^{2}\left(t\right), \end{array}$$

where

$$Q\left(t\right)={\left[Q_{1}\left(t\right),Q_{2}\left(t\right),\ldots ,Q_{U}\left(t\right)\right]}^{T}$$

is the aggregate UAV queue-state vector.

The conditional one-step Lyapunov drift is given by

(59) $$\begin{array}{c} \Delta \left(Q\left(t\right)\right)=E\left[L(Q(t+1))-L(Q(t))|Q(t)\right]. \end{array}$$

Minimising the Lyapunov drift encourages queue stability by preventing unbounded backlog growth. However, in disaster-response missions, queue stability alone is insufficient because the system must also minimise delay, energy consumption, and information staleness. Therefore, a drift-plus-penalty formulation is used.

### 3.4. Drift-Plus-Penalty Objective

Let $g_{i}\left(t\right)$ denote the instantaneous cost associated with the selected processing mode for UAV *i*. This cost combines energy consumption, processing delay, and AoI:

(60) $$\begin{array}{c} g_{i}\left(t\right)=\alpha E_{i}\left(t\right)+\beta T_{i}\left(t\right)+\gamma \Delta_{i}\left(t\right), \end{array}$$

When UAV *i* evaluates the processing mode for a specific collected task *j*, the UAV-level instantaneous cost $g_{i}\left(t\right)$ is instantiated by the corresponding task- and mode-specific cost $g_{ij}^{m}\left(t\right)$, as defined in the following subsection. The $E_{i}\left(t\right)$ is the energy consumed by the selected mode, $T_{i}\left(t\right)$ is the corresponding task completion delay, $\Delta_{i}\left(t\right)$ is the resulting AoI-related cost, and α, β, and γ are weighting coefficients.

The drift-plus-penalty objective is then written as

(61) $$\begin{array}{c} \Delta \left(Q\left(t\right)\right)+VE\left[\sum_{i=1}^{U}g_{i}\left(t\right)|Q\left(t\right)\right], \end{array}$$

where V>0 is the Lyapunov control parameter. A larger value of *V* places greater emphasis on reducing energy–delay–AoI cost, while a smaller value of *V* prioritises queue reduction and system responsiveness.

Starting from the queue update,

(62) $$Q_{i}\left(t+1\right)=\max\;\left[Q_{i}\left(t\right)-\mu_{i}\left(t\right),0\right]+A_{i}\left(t\right),$$

and using ${\left(\max[x,0]\right)}^{2}\leq x^{2}$, we obtain

(63) $$\begin{array}{cc} Q_{i}^{2}\left(t+1\right) & ={\left(\max\left[Q_{i}\left(t\right)-\mu_{i}\left(t\right),0\right]+A_{i}\left(t\right)\right)}^{2}\\ & \leq Q_{i}^{2}\left(t\right)+\mu_{i}^{2}\left(t\right)+A_{i}^{2}\left(t\right)+2Q_{i}\left(t\right)\left(A_{i}\left(t\right)-\mu_{i}\left(t\right)\right). \end{array}$$

Therefore,

(64) $$Q_{i}^{2}\left(t+1\right)-Q_{i}^{2}\left(t\right)\leq \mu_{i}^{2}\left(t\right)+A_{i}^{2}\left(t\right)+2Q_{i}\left(t\right)\left(A_{i}\left(t\right)-\mu_{i}\left(t\right)\right).$$

Summing (64) over all UAVs, multiplying by 1/2, and conditioning on the current queue state gives

(65) $$\begin{array}{cc} \Delta (Q(t)) & \leq \frac{1}{2}\sum_{i=1}^{U}E\left[A_{i}^{2}\left(t\right)+\mu_{i}^{2}\left(t\right)|Q\left(t\right)\right]\\ & +\sum_{i=1}^{U}Q_{i}\left(t\right)E\left[A_{i}\left(t\right)-\mu_{i}\left(t\right)|Q\left(t\right)\right]. \end{array}$$

Under Assumption 1,

(66) $$A_{i}^{2}\left(t\right)+\mu_{i}^{2}\left(t\right)\leq {\left(A_{i}^{\max}\right)}^{2}+{\left(\mu_{i}^{\max}\right)}^{2}.$$

Hence, the finite drift constant is explicitly defined as

(67) $$B≜\frac{1}{2}\sum_{i=1}^{U}\left[{\left(A_{i}^{\max}\right)}^{2}+{\left(\mu_{i}^{\max}\right)}^{2}\right].$$

The conditional drift is, consequently, bounded by

(68) $$\Delta \left(Q\left(t\right)\right)\leq B+\sum_{i=1}^{U}Q_{i}\left(t\right)E\left[A_{i}\left(t\right)-\mu_{i}\left(t\right)|Q\left(t\right)\right].$$

Adding the weighted conditional penalty yields

(69) $$\begin{array}{cc} \; & \Delta \left(Q\left(t\right)\right)+VE\left[g(t)|Q(t)\right]\\ \; & \leq B+\sum_{i=1}^{U}Q_{i}\left(t\right)E\left[A_{i}\left(t\right)-\mu_{i}\left(t\right)|Q\left(t\right)\right]+VE\left[g(t)|Q(t)\right]. \end{array}$$

At each decision slot, the proposed controller selects the feasible local or MEC mode that minimises the decision-dependent terms on the right-hand side of (69).

### 3.5. Per-Task Binary Offloading Decision

For each task *j* collected by UAV *i*, the controller evaluates two candidate processing modes: local processing and MEC offloading. The local mode is feasible only if its processing delay and energy consumption satisfy the task deadline and residual UAV-energy constraints:

(70) $$\begin{array}{cccc} T_{ij}^{\mathrm{loc}}\left(t\right) & \leq \tau_{j},\; & E_{ij}^{\mathrm{loc}}\left(t\right) & \leq E_{i}^{\mathrm{rem}}\left(t\right). \end{array}$$

Similarly, MEC offloading is feasible only if the wireless link is available and the total MEC-assisted processing delay and transmission energy satisfy

(71) $$R_{i}\left(t\right)>0,$$

(72) $$T_{ij}^{\mathrm{mec}}\left(t\right)=T_{ij}^{\mathrm{tx}}\left(t\right)+T_{j}^{\mathrm{edge}}\leq \tau_{j},$$

(73) $$E_{ij}^{\mathrm{mec}}\left(t\right)=E_{ij}^{\mathrm{tx}}\left(t\right)\leq E_{i}^{\mathrm{rem}}\left(t\right).$$

Here, $E_{i}^{\mathrm{rem}}\left(t\right)$ is the residual energy of UAV *i* after accounting for flight and previously processed tasks. Let $M_{ij}\left(t\right)\subseteq \left\{\mathrm{loc},\mathrm{mec}\right\}$ denote the set of feasible processing modes for task *j*.

For each feasible mode $m\in M_{ij}\left(t\right)$, the instantaneous task cost is defined as

(74) $$\begin{array}{c} g_{ij}^{m}\left(t\right)=\alpha E_{ij}^{m}\left(t\right)+\beta T_{ij}^{m}\left(t\right)+\gamma \Delta_{ij}^{m}\left(t\right), \end{array}$$

where $E_{ij}^{m}\left(t\right)$, $T_{ij}^{m}\left(t\right)$, and $\Delta_{ij}^{m}\left(t\right)$ are the energy consumption, completion delay, and completion-time AoI associated with mode *m*, respectively.

Because each UAV maintains one aggregate processing queue, the candidate queue state remains UAV-indexed:

(75) $$\begin{array}{c} Q_{i}^{m}\left(t+1\right)=\max\left[Q_{i}\left(t\right)-\mu_{i}^{m}\left(t\right),0\right]+A_{i}\left(t\right). \end{array}$$

The corresponding one-step quadratic drift term is

(76) $$\begin{array}{c} D_{i}^{m}\left(t\right)=\frac{1}{2}\left[{\left(Q_{i}^{m}\left(t+1\right)\right)}^{2}-Q_{i}^{2}\left(t\right)\right]. \end{array}$$

The Lyapunov objective associated with task *j* and mode *m* is

(77) $$\begin{array}{c} J_{ij}^{m}\left(t\right)=D_{i}^{m}\left(t\right)+Vg_{ij}^{m}\left(t\right). \end{array}$$

The implementation evaluates the exact one-step quadratic queue difference for each feasible candidate mode. Terms that are independent of the current local/MEC decision can be omitted without changing the selected minimiser.

The implemented objective in (77) should be distinguished from the conditional upper-bound expression in (69). The analytical derivation uses the standard conditional drift-plus-penalty upper bound to characterise long-term queue and cost behaviour, whereas the event-driven implementation evaluates the realised one-step quadratic queue difference for each feasible LOCAL/MEC candidate at the task-collection epoch. Both follow the same Lyapunov principle of balancing queue reduction against the weighted energy–delay–AoI penalty, but the realised one-step objective is not claimed to be algebraically identical to the conditional upper bound for every queue state and action. Accordingly, the analytical performance result below applies to the corresponding bound-minimising drift-plus-penalty policy under its stated assumptions, while the reported simulation results characterise the implemented one-step decision rule.

When at least one processing mode is feasible, the selected mode is

(78) $$\begin{array}{c} m_{ij}^{★}\left(t\right)=\arg\min_{m\in M_{ij}\left(t\right)}J_{ij}^{m}\left(t\right). \end{array}$$

The corresponding binary offloading decision is

(79) $$\begin{array}{c} x_{ij}^{★}\left(t\right)=\left\{\begin{array}{cc} 0,\; & m_{ij}^{★}\left(t\right)=\mathrm{loc},\\ 1,\; & m_{ij}^{★}\left(t\right)=\mathrm{mec}. \end{array}\right. \end{array}$$

If $M_{ij}\left(t\right)=⌀$, neither local processing nor MEC offloading satisfies the deadline, communication, and residual-energy constraints, and task *j* is marked as dropped.

This decision rule selects the feasible processing mode with the minimum drift-plus-penalty objective at the task-collection time. MEC is, therefore, retained as a selective processing option rather than being assumed to be universally superior to local execution. It becomes more likely to be selected when the wireless channel supports a sufficiently high transmission rate, the combined transmission and edge-processing delay satisfies the task deadline, and offloading provides a lower energy–delay–AoI and queue-backlog cost than local processing. Conversely, local execution is preferred when onboard processing is feasible and efficient or when weak channel conditions make MEC offloading unfavourable.

**Figure 2:**
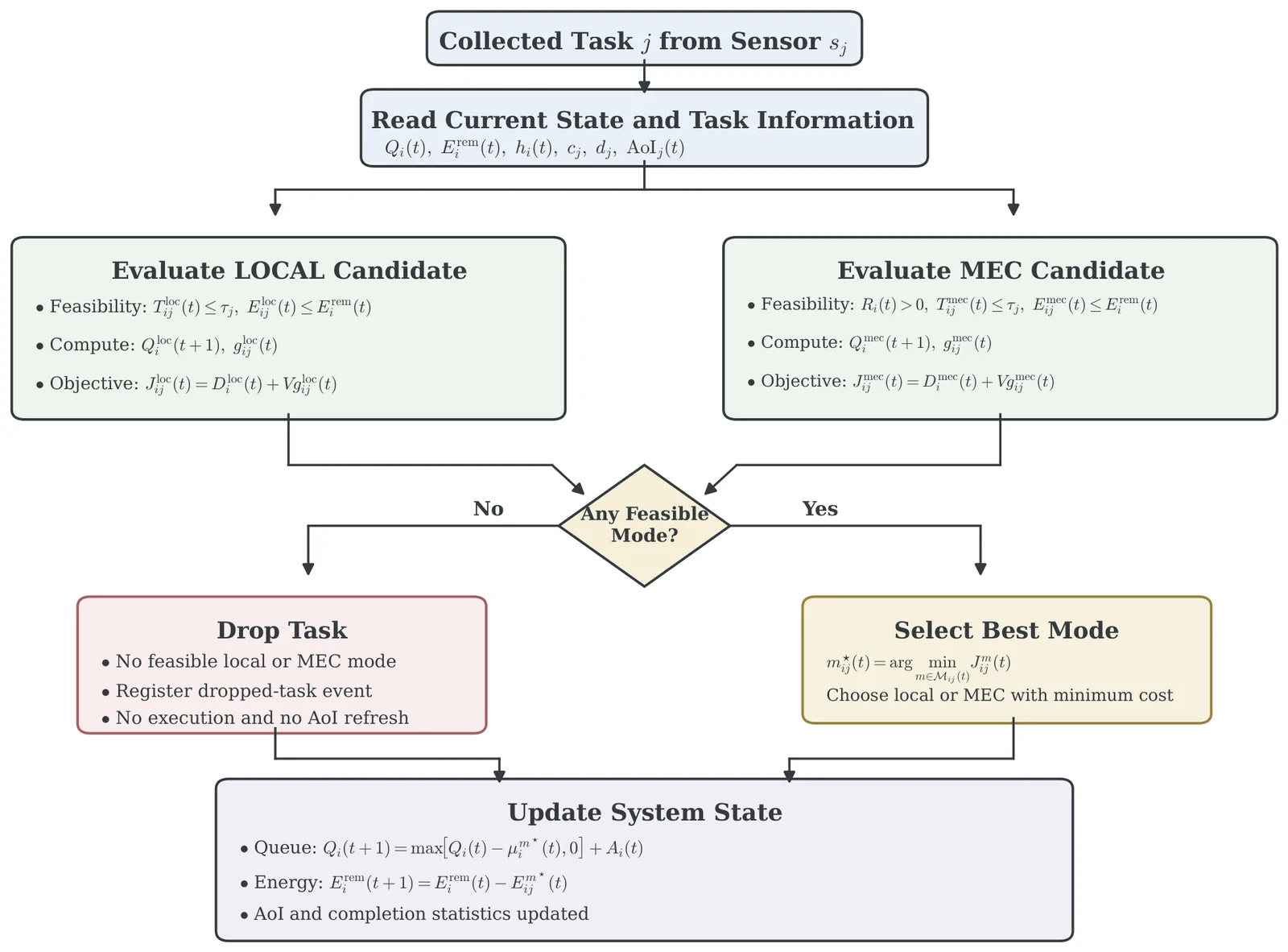
Offloading layer for temporal processing decisions.

Figure 2 illustrates the role of the Lyapunov-based binary offloading layer. After task collection, this layer makes the temporal processing decision by selecting either local UAV processing or MEC offloading according to queue backlog, delay, energy, and processing feasibility.

### 3.6. Local Processing Cost

For local processing, the execution delay of task *j*, collected by UAV *i*, is

(80) $$\begin{array}{c} T_{ij}^{\mathrm{loc}}\left(t\right)=\frac{C_{j}}{f_{i}^{\mathrm{uav}}}, \end{array}$$

where $C_{j}=d_{j}c_{j}$ is the number of CPU cycles required by task *j*, and $f_{i}^{\mathrm{uav}}$ is the onboard CPU capacity of UAV *i*.

The corresponding local-computation energy is

(81) $$\begin{array}{c} E_{ij}^{\mathrm{loc}}\left(t\right)=P_{i}^{\mathrm{cpu}}T_{ij}^{\mathrm{loc}}\left(t\right), \end{array}$$

where $P_{i}^{\mathrm{cpu}}$ is the computation-power coefficient of UAV *i*.

The completion-time AoI under local processing is

(82) $$\begin{array}{c} \Delta_{ij}^{\mathrm{loc}}\left(t\right)=t_{ij}^{\mathrm{col}}-g_{j}+T_{ij}^{\mathrm{loc}}\left(t\right), \end{array}$$

where $t_{ij}^{\mathrm{col}}$ is the time at which UAV *i* first collects the update associated with sensor *j*, and $g_{j}$ is the generation or availability time of that update.

### 3.7. MEC-Offloading Cost

For MEC offloading, the uplink transmission rate from UAV *i* to the MEC node is

(83) $$\begin{array}{c} R_{i}\left(t\right)=B_{i}\left(t\right)\log_{2}\left(1+\frac{P_{i}\left(t\right)h_{i}\left(t\right)}{\sigma_{i}^{2}\left(t\right)+I_{i}\left(t\right)}\right), \end{array}$$

where $B_{i}\left(t\right)$ is the available bandwidth, $P_{i}\left(t\right)$ is the transmission power, $h_{i}\left(t\right)$ is the channel gain, $\sigma_{i}^{2}\left(t\right)$ is the noise power, and $I_{i}\left(t\right)$ is the interference power.

The transmission delay for task *j* is

(84) $$\begin{array}{c} T_{ij}^{\mathrm{tx}}\left(t\right)=\frac{d_{j}}{R_{i}\left(t\right)}, \end{array}$$

where $d_{j}$ is the data size of task *j*.

The MEC-side processing delay is

(85) $$\begin{array}{c} T_{j}^{\mathrm{edge}}=\frac{C_{j}}{f^{\mathrm{mec}}}, \end{array}$$

where $f^{\mathrm{mec}}$ is the CPU capacity of the MEC node.

Therefore, the total MEC-assisted processing delay is

(86) $$\begin{array}{c} T_{ij}^{\mathrm{mec}}\left(t\right)=T_{ij}^{\mathrm{tx}}\left(t\right)+T_{j}^{\mathrm{edge}}. \end{array}$$

The transmission energy consumed by UAV *i* when offloading task *j* is

(87) $$\begin{array}{c} E_{ij}^{\mathrm{mec}}\left(t\right)=E_{ij}^{\mathrm{tx}}\left(t\right)=P_{i}\left(t\right)T_{ij}^{\mathrm{tx}}\left(t\right). \end{array}$$

The completion-time AoI under MEC offloading is

(88) $$\begin{array}{c} \Delta_{ij}^{\mathrm{mec}}\left(t\right)=t_{ij}^{\mathrm{col}}-g_{j}+T_{ij}^{\mathrm{mec}}\left(t\right), \end{array}$$

where $T_{ij}^{\mathrm{mec}}\left(t\right)$ includes both the uplink transmission delay and the MEC-side processing delay.

### 3.8. Conditional Queue-Stability Result

The following result formalises the performance of the proposed drift-plus-penalty controller under the boundedness and feasibility conditions stated in Section 3.2.

**Theorem 1.**  *Suppose that Assumptions 1–4 hold, V>0, and E[L(Q(0))]<∞. For the analytical drift-plus-penalty policy that minimises the decision-dependent right-hand side of *(69)* over the feasible local and MEC actions at every decision slot, the following conditional performance bounds hold:*

*1.*  *The achieved time-average cost satisfies*
  (89) $${\bar{g}}^{\mathrm{LBO}}\leq g^{★}+\frac{B}{V},$$
  *where B is explicitly defined in *(67)*.*
*2.*  *The task queues are strongly stable, and their time-average expected aggregate backlog satisfies*
(90) $$\underset{T\to \infty }{lim sup}\frac{1}{T}\sum_{t=0}^{T-1}\sum_{i=1}^{U}E\left[Q_{i}\left(t\right)\right]\leq \frac{B+V\left(g^{\epsilon }-g_{\min}\right)}{\epsilon }.$$

**Proof.**  The complete proof is provided in Appendix A. The proof first derives the quadratic drift bound and the explicit constant *B*. It then compares the proposed per-slot policy with the stationary cost-optimal policy $\pi^{★}$ to establish (89). Finally, comparison with the ϵ-slack stabilising policy $\pi^{\epsilon }$ establishes (90).   □

Equation (89) provides the standard conditional O(1/V) cost-optimality bound, while Equation (90) provides the corresponding conditional O(V) backlog bound under the stated assumptions. Accordingly, increasing *V* places greater emphasis on the energy–delay–AoI penalty, whereas decreasing *V* places greater emphasis on queue reduction. The theorem is conditional on the boundedness, feasibility, and stationary-policy assumptions stated above. The finite-horizon queue traces reported in the simulation section provide empirical evidence of bounded mission-duration behaviour, but they are not used as an independent proof of infinite-horizon strong stability. The theorem, therefore, characterises the analytical bound-minimising drift-plus-penalty policy. The event-driven MATLAB R2025a implementation uses the realised one-step objective in (77); consequently, the theorem is not presented as an independent proof of strong stability or the O(1/V) optimality bound for the finite-horizon implementation itself. Instead, it provides the theoretical Lyapunov reference underlying the temporal-control design, while the implemented queue behaviour is evaluated empirically in Section 6.

### 3.9. Summary

The Lyapunov-based optimisation framework provides the temporal decision layer of the proposed multi-UAV system. Unlike formulations that optimise CPU frequency, partitioning ratios, or multiple MEC server selection, the proposed model adopts a simplified binary decision structure. Each collected task is either processed locally or fully offloaded to a single MEC node, while infeasible tasks are dropped. This simplification improves interpretability and aligns the temporal layer with the overall layered framework: the spatial layer determines where and when data are collected, the temporal layer determines how collected tasks are processed, and the safety layer monitors conflict-free multi-UAV execution.

### 3.10. Interaction with the 3D-TSPN–GA–RRT-Connect Framework

The Lyapunov-based optimisation (LBO) module operates at the temporal execution stage to make queue-aware computation and binary MEC-offloading decisions, while the 3D-TSPN–GA–RRT-Connect framework governs the spatial planning layer by determining the sensing-neighbourhood visitation sequence, guaranteeing coverage, and generating geometrically feasible obstacle-aware trajectories.

The interaction between the temporal and spatial layers is achieved through shared system states. Specifically, the spatial layer determines the collection time of each sensor, which directly affects the resulting Age of Information (AoI), task-arrival process, and queue evolution. The AoI-aware genetic algorithm (GA) uses estimated visitation times to prioritise freshness-aware routing, while RRT-Connect ensures that each inter-neighbourhood transition is feasible in the 3D-obstacle environment. After each collection event, the LBO module evaluates the current queue backlog, remaining UAV energy, wireless channel quality, and UAV–MEC distance to select either local processing or full offloading to the MEC node.

This coupling establishes a structured spatial–temporal optimisation framework in which trajectory planning, sensing freshness, queue stability, and computation offloading are coordinated during mission execution, while preserving a clear separation between the spatial planning and temporal control layers.

## 4. Layered Multi-UAV Framework

This section presents the proposed layered architecture integrating 3D-TSPN-based coverage modelling, AoI-aware genetic algorithm (GA) sequencing, RRT-Connect path feasibility checking, inflated obstacle avoidance, and multi-UAV safety monitoring. The framework is designed to operate reliably in complex disaster environments while maintaining a clear separation between spatial planning, temporal queue-aware optimisation, and safety monitoring.

### 4.1. Framework Overview

**Figure 3:**
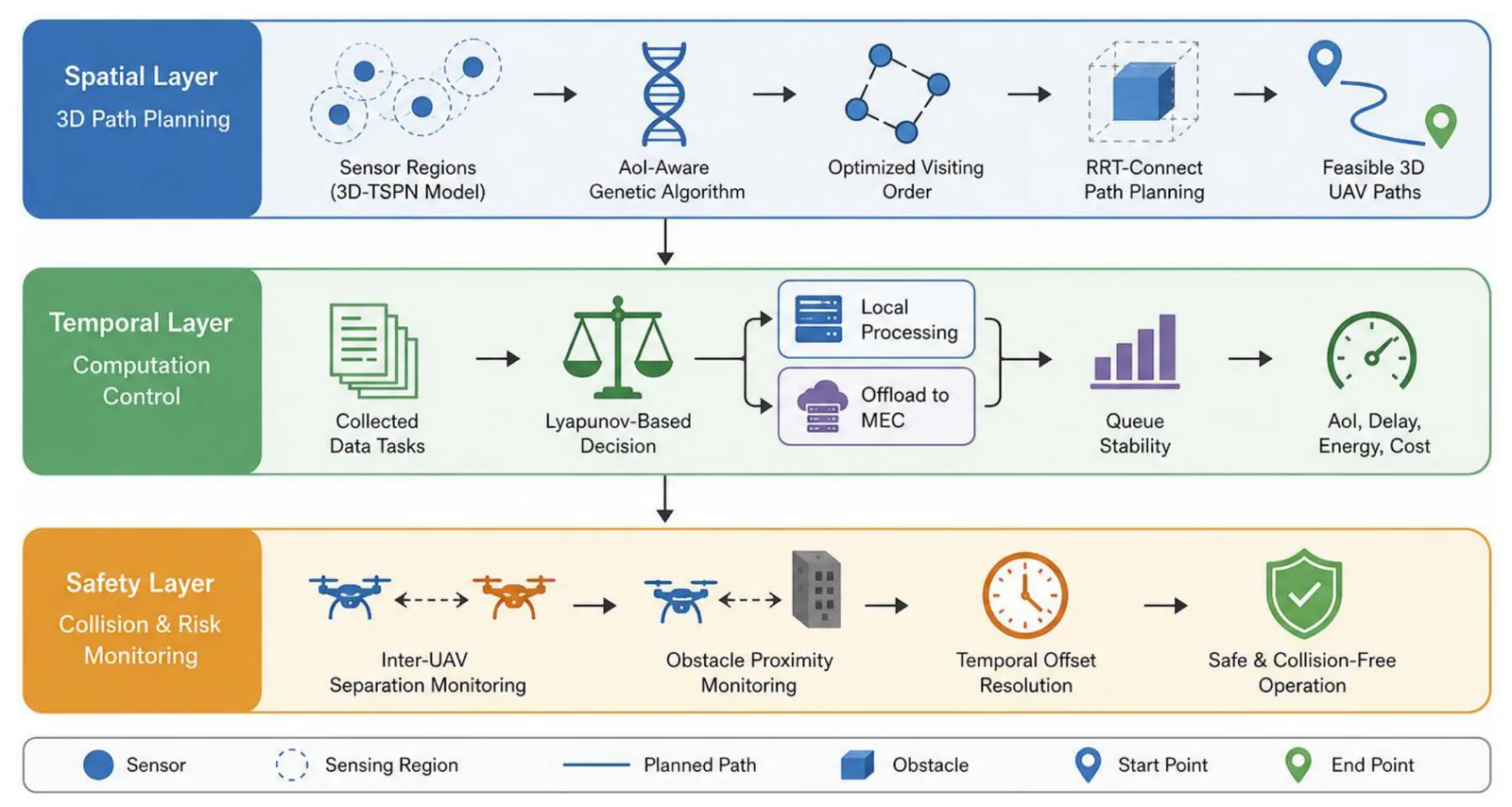
Spatial, temporal, and safety layers of the proposed multi-UAV framework.

Figure 3 presents the three interacting layers of the proposed framework. The spatial layer performs balanced sensing-zone assignment, 3D-TSPN-based coverage modelling, AoI-aware sensor sequencing, and obstacle-aware path generation. The temporal layer makes queue-aware binary decisions between local processing and MEC offloading. The safety layer validates obstacle clearance and inter-UAV separation and applies conflict-resolution actions when required. Although the layers have distinct optimisation responsibilities, they exchange collection times, queue states, wireless conditions, energy states, and validated trajectories during mission execution.

The proposed system consists of five coordinated phases:

1. **Coverage Modelling using 3D-TSPN:** Each ground sensor is represented by a 3D sensing neighbourhood, allowing the UAV to collect data without visiting the exact sensor coordinate.
2. **AoI-Aware GA-Based Sequencing:** An AoI-aware GA computes the visitation order of the assigned sensor neighbourhoods by considering flight time, cumulative AoI, coverage feasibility, and path smoothness.
3. **RRT-Connect Path Feasibility Checking:** For each leg in the GA solution, RRT-Connect is used to verify and generate feasible obstacle-aware 3D paths between sensing neighbourhoods.
4. **Path Post-Processing:** Generated paths are simplified, redundant waypoints are removed, and the final trajectories are resampled to support smooth and feasible UAV movement.
5. **Multi-UAV Safety Monitoring:** Continuous-time conflict detection, closest-approach distance checking, obstacle inflation margins, and temporal desynchronisation are used to support safe simultaneous UAV operation.

This layered structure enables robust global–local coordination while avoiding unnecessary algorithmic complexity.

**Figure 4:**
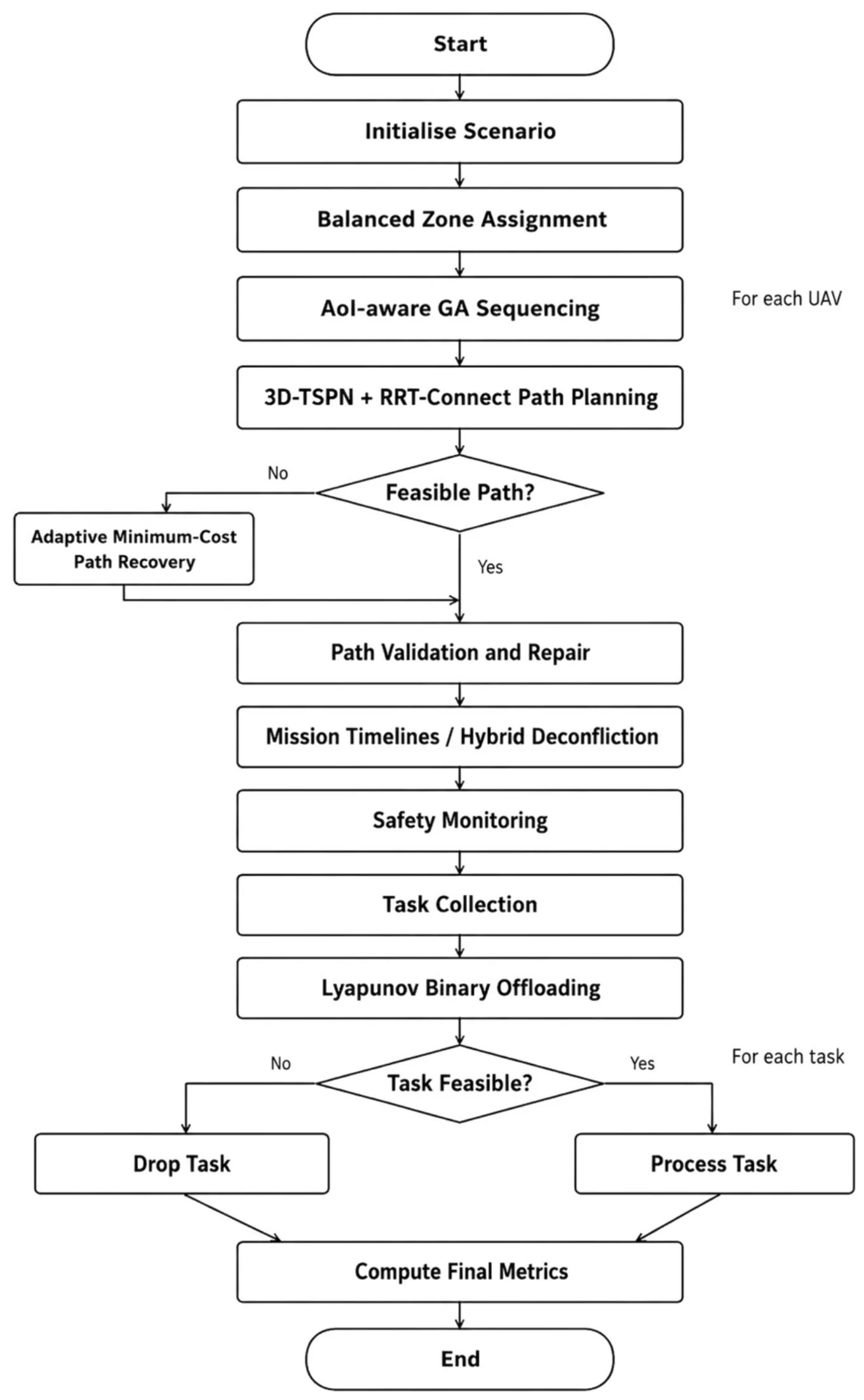
Proposed multi-UAV optimisation process.

Figure 4 illustrates the overall workflow of the proposed multi-UAV optimisation framework. The process begins with scenario initialisation and balanced zone assignment, after which an AoI-aware genetic algorithm determines the sensing-neighbourhood visitation sequence for each UAV. The 3D-TSPN model and RRT-Connect planner then generate an obstacle-aware three-dimensional route. If the resulting route is infeasible, the adaptive minimum-cost path recovery module generates and evaluates alternative coverage-preserving routes before returning the selected feasible solution to the main workflow.

The resulting trajectories are subsequently subjected to path validation and repair, followed by mission-timeline construction and hybrid inter-UAV deconfliction. The safety-monitoring layer then verifies obstacle clearance and continuous-time inter-UAV separation before task collection. For each collected task, the Lyapunov-based binary offloading controller selects either local processing or MEC offloading according to queue backlog, delay, energy, AoI, wireless-link conditions, and processing feasibility. Tasks for which neither processing mode is feasible are dropped, whereas feasible tasks are processed before the final mission-performance metrics are computed.

Algorithm 1 provides the complete execution sequence of the proposed framework. Steps 1–7 describe the spatial planning, route-recovery, and fleet-level safety stages. The subsequent steps identify the first valid sensor-collection events and apply the Lyapunov-based binary processing controller to each collected task. The algorithm concludes with the final coverage, processing, obstacle, and continuous-time safety audits. Unlike the workflow diagram, which illustrates the relationships among the framework components, Algorithm 1 specifies their computational order, conditional branches, state updates, and links to the corresponding optimisation constraints and objectives.
**Algorithm 1:** Complete Layered Multi-UAV Planning and Processing Procedure   **Input:** Map A; UAV set U; sensor set S; obstacle set O; sensing radius $R_{s}$; minimum separation $d_{\min}$; battery limits; and GA, RRT-Connect, MEC, Lyapunov, and safety parameters.   **Output:** Validated UAV trajectories; LOCAL/MEC processing decisions; queue and residual-energy states; AoI values; safety outcomes; and mission-validity metrics.**1** Initialise the UAV states, sensors, obstacles, MEC node, queues, batteries, and simulation parameters;**2** Partition the sensors into balanced UAV service zones and determine $a_{ij}$ subject to (38);
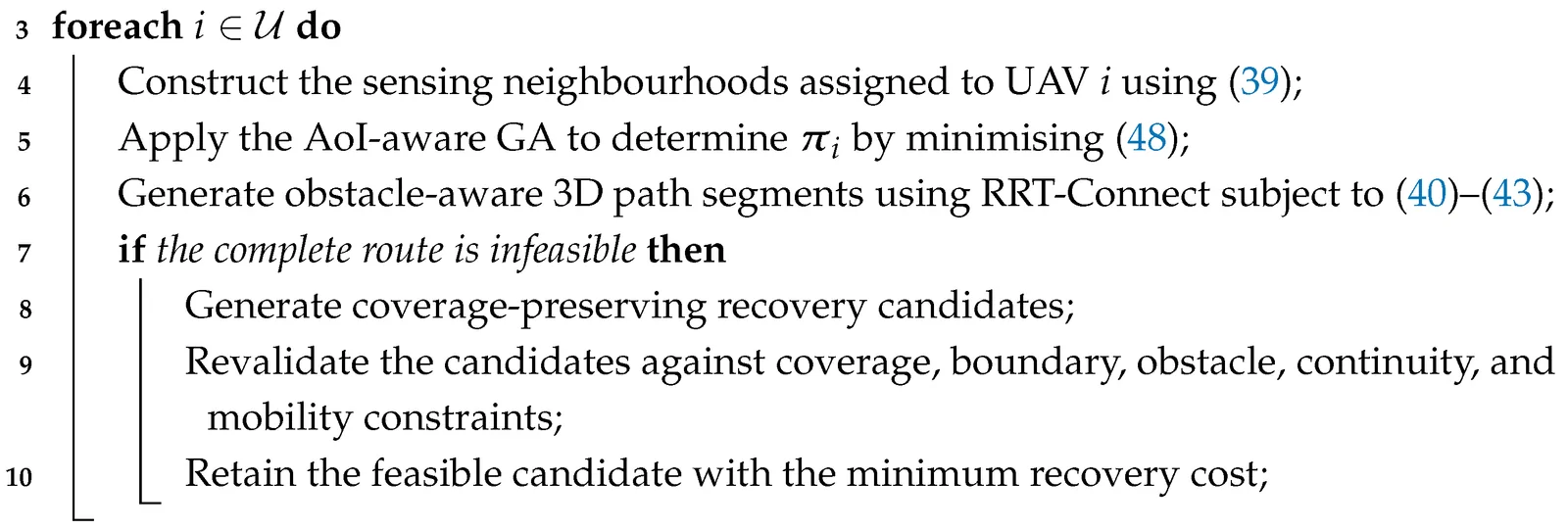
**11** Validate all UAV routes against sensing coverage, map boundaries, inflated obstacles, path continuity, and prescribed endpoints;**12** Construct the UAV mission timelines and evaluate continuous-time inter-UAV separation;
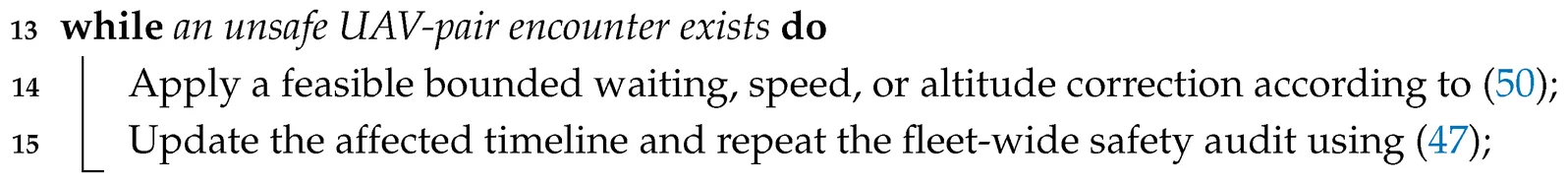
**16** Identify the first valid entry into each sensing neighbourhood and record the collection events;
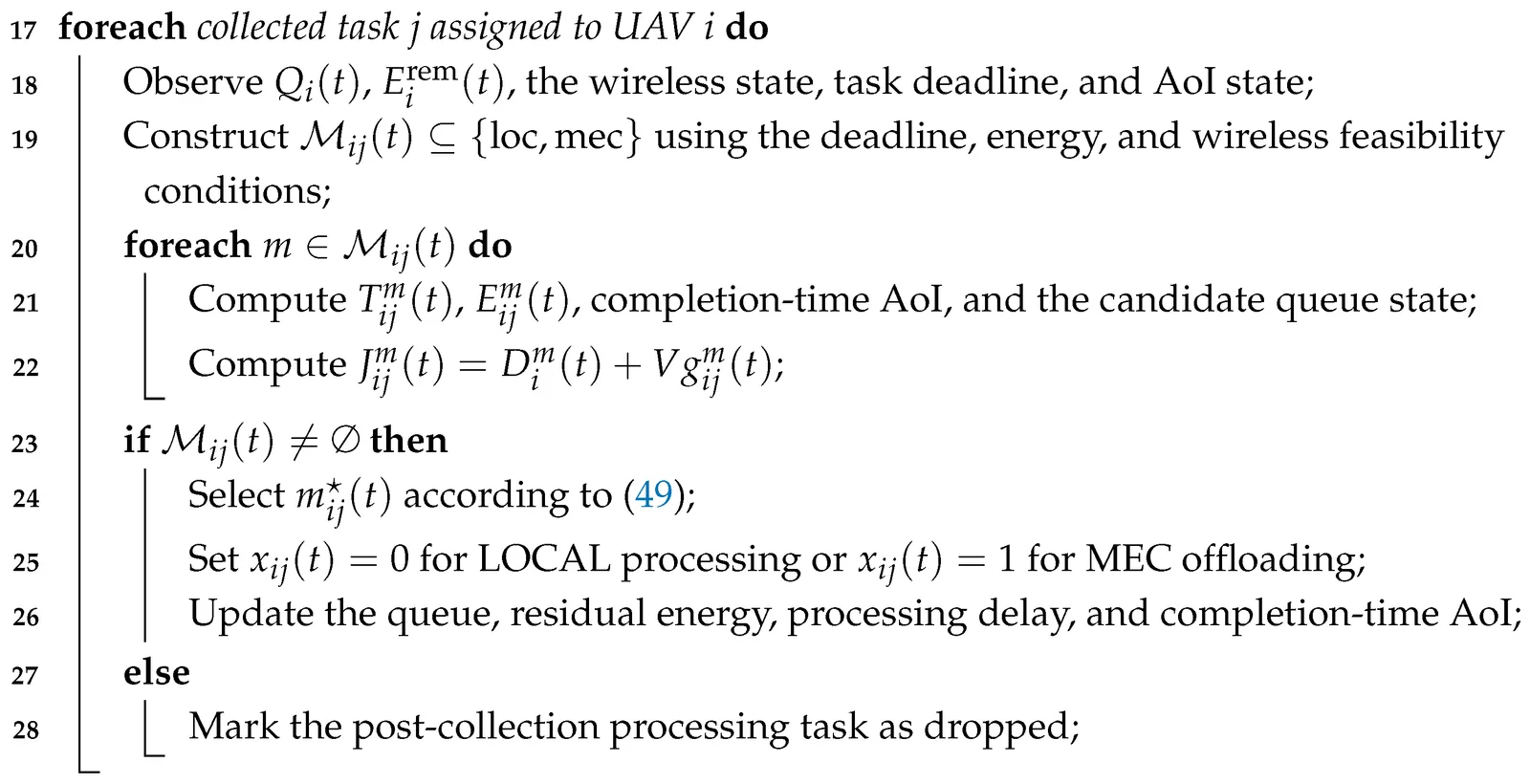
**29** Perform the final coverage, processing, obstacle-clearance, and continuous-time all-pair safety audits;**30** Compute mission completion time, AoI, energy consumption, queue backlog, LOCAL/MEC rates, dropped-task count, and minimum UAV separation;**31** **return** the validated trajectories, processing decisions, system states, and final performance metrics;

### 4.2. Balanced Zone Assignment

Before route sequencing, the sensing nodes are partitioned into balanced spatial zones and each zone is assigned to one UAV [38,39,40]. The objective is to distribute the sensing workload approximately evenly among the UAVs while preserving spatial locality. This assignment reduces unnecessary route overlap and prevents a single UAV from receiving a disproportionately large sensing workload. The balanced zone-assignment step is performed during mission initialisation before the AoI-aware GA is applied independently to the sensing neighbourhoods assigned to each UAV.

### 4.3. 3D-TSPN Coverage Formulation

As defined in Section 2, each sensing region is represented by a sphere:

$$N_{j}=\left\{x:∥x-s_{j}∥\leq R_{s}\right\}.$$

The objective is to find an ordered sequence

$$\Pi =(n_{1},n_{2},\ldots ,n_{N})$$

such that each sensor neighbourhood is visited at least once while minimising the spatial mission cost. The 3D-TSPN formulation is expressed as follows:

(91) $$\begin{array}{c} \min_{\Pi }\;\sum_{k=1}^{N-1}d\left(N_{\Pi_{k}},N_{\Pi_{k+1}}\right), \end{array}$$

subject to:

(92) $$\begin{array}{c} p_{i}\left(t_{k}\right)\in N_{\Pi_{k}},\;\forall k\in \left\{1,\ldots ,N\right\}. \end{array}$$

Unlike conventional TSP formulations, the UAV is not required to visit the exact sensor coordinate. Instead, it only needs to enter the corresponding sensing neighbourhood. This provides additional spatial flexibility around obstacles and enables RRT-Connect to select feasible approach points within each neighbourhood.

**Figure 5:**
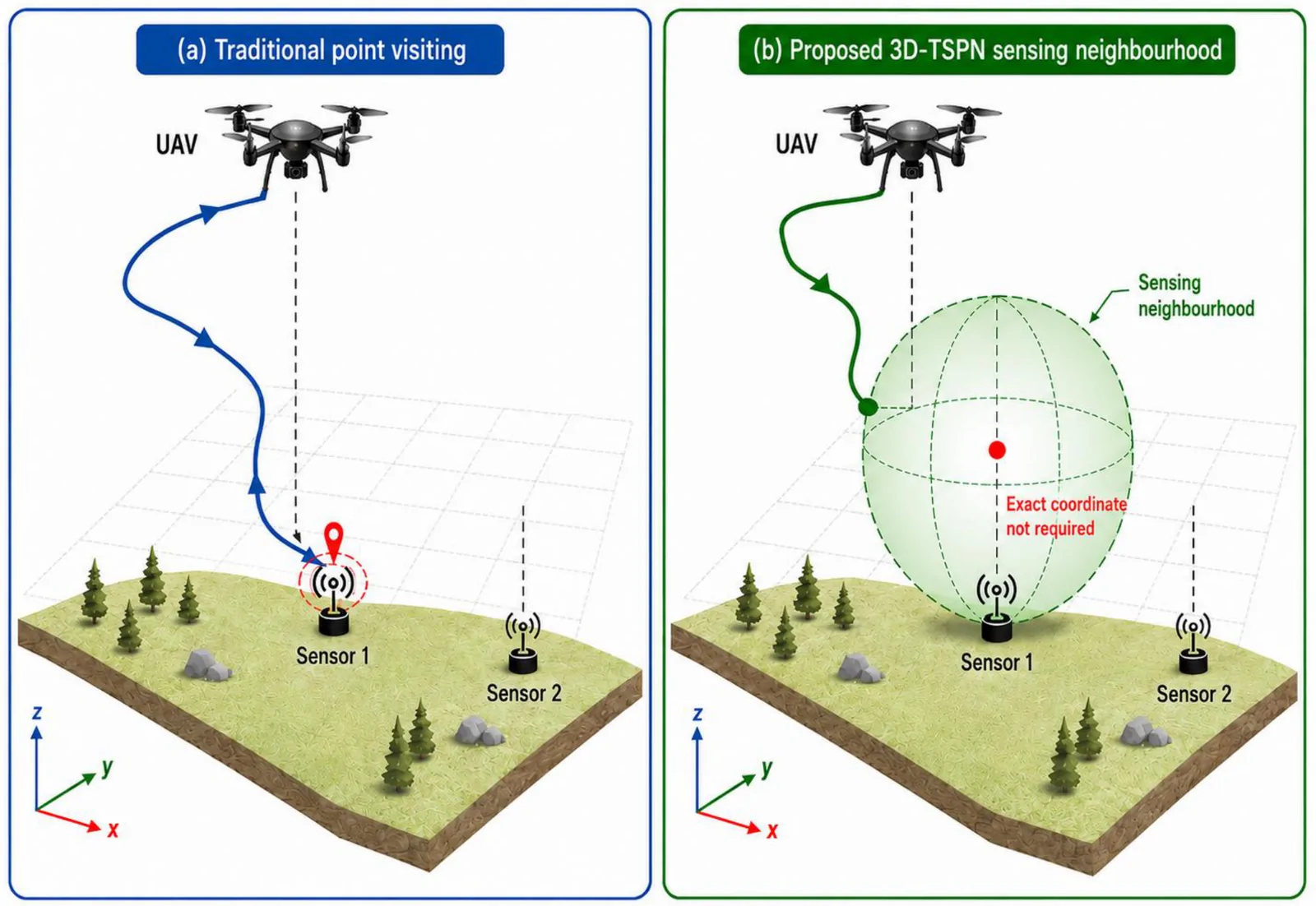
3D-TSPN sensing neighbourhood-based data collection compared with traditional exact-point sensor visiting.

Figure 5 illustrates that, unlike traditional point visiting, the proposed 3D-TSPN model allows the UAV to collect data once it enters the sensing neighbourhood, without visiting the exact sensor coordinate.

### 4.4. Genetic Algorithm Representation

In the proposed spatial layer, each GA chromosome is represented as a permutation of the assigned TSPN neighbourhood indices:

$$c=\left[c_{1},c_{2},\ldots ,c_{M}\right],$$

where *M* is the number of sensors assigned to the corresponding UAV. Each gene corresponds to one unique sensing neighbourhood, and the gene order defines the UAV visitation sequence. To preserve valid TSPN routing, repeated genes are not allowed.

The initial population of size *P* is generated using a mixed strategy combining random permutations, nearest-neighbour sequences, and greedy-randomised routes. This initialisation improves early solution quality while maintaining population diversity. In addition, AoI-related priority is reflected through the fitness function, enabling the GA to favour routes that reduce information staleness during mission execution.

### 4.5. AoI-Aware GA Fitness Function

The GA fitness function is designed to optimise the spatial layer only. It evaluates each candidate route according to flight time, cumulative AoI, and path smoothness. Computation energy, queue stability, and local/MEC-offloading decisions are handled separately by the Lyapunov-based temporal layer.

For a chromosome c, the implemented fitness function is defined as

(93) $$\begin{array}{c} F\left(c\right)=w_{d}T_{\mathrm{fly}}\left(c\right)+w_{a}\Delta_{\mathrm{AoI}}\left(c\right)+w_{s}S_{\mathrm{smooth}}\left(c\right), \end{array}$$

where $w_{d}$, $w_{a}$, and $w_{s}$ are non-negative weighting coefficients associated with flight time, AoI, and trajectory smoothness, respectively.

The flight-time term is computed as

(94) $$\begin{array}{c} T_{\mathrm{fly}}\left(c\right)=\frac{D_{\mathrm{path}}\left(c\right)}{v_{\mathrm{uav}}}, \end{array}$$

where $D_{\mathrm{path}}\left(c\right)$ is the total length of the RRT-Connect-generated route and $v_{\mathrm{uav}}$ is the UAV speed.

The cumulative AoI term is approximated using the estimated arrival time at each sensing neighbourhood:

(95) $$\begin{array}{c} \Delta_{\mathrm{AoI}}\left(c\right)=\sum_{j=1}^{M}t_{\Pi_{j}}, \end{array}$$

where $t_{\Pi_{j}}$ denotes the estimated time at which the UAV first enters the sensing neighbourhood of sensor $\Pi_{j}$. This term encourages the GA to prioritise routes that collect time-sensitive data earlier.

The smoothness term penalises abrupt changes in flight direction:

(96) $$\begin{array}{c} S_{\mathrm{smooth}}\left(c\right)=\frac{1}{K}\sum_{k=1}^{K}\theta_{k}, \end{array}$$

where $\theta_{k}$ is the turning angle between consecutive path segments and *K* is the number of evaluated turns.

Geometrically infeasible chromosomes are handled through hard rejection rather than a finite weighted penalty. Specifically, if a candidate path cannot be generated, contains fewer than two valid waypoints, or fails to cover any assigned sensing neighbourhood, it is excluded from the feasible candidate set by assigning

(97) $$\begin{array}{c} F(c)=\infty . \end{array}$$

This also covers failed RRT-Connect transitions, since a chromosome is only evaluated after an obstacle-aware feasible path has been generated. Consequently, infeasible candidates cannot be selected as valid GA solutions.

This simplified fitness structure ensures that the GA remains focused on spatial route quality, while temporal computation decisions are made independently by the Lyapunov controller.

**Figure 6:**
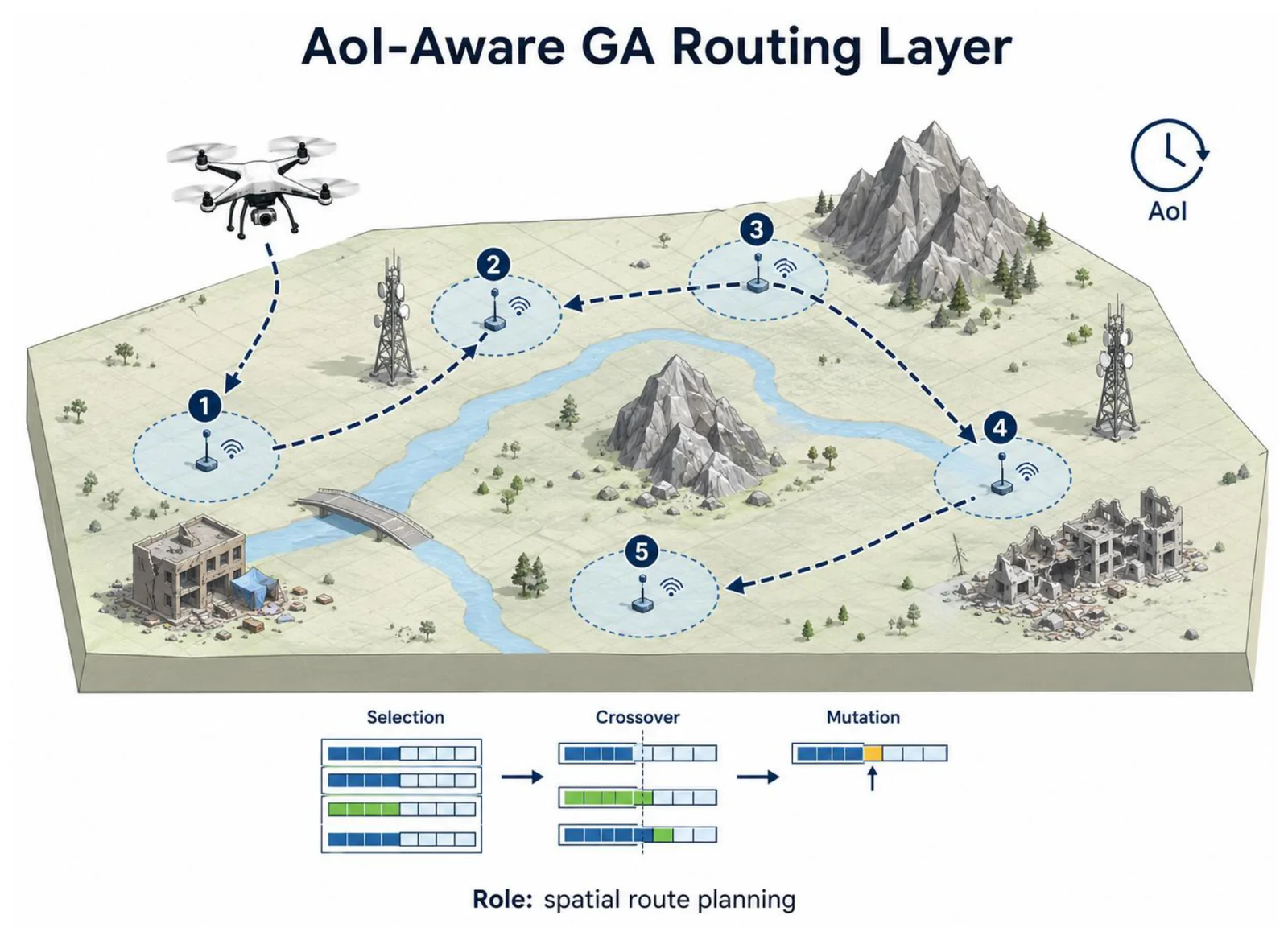
GA routing layer for spatial route planning. The circled numbers indicate the GA-determined visitation order of the sensing neighbourhoods.

Figure 6 illustrates the role of the AoI-aware GA routing layer in the proposed framework. This layer focuses on spatial route planning by determining the UAV visiting sequence across sensing neighbourhoods while considering AoI, obstacles, and route feasibility.

### 4.6. GA Operators

To efficiently explore the permutation-based 3D-TSPN search space while preserving valid route structures, the GA employs selection, crossover, mutation, and elitism operators.

#### 4.6.1. Selection

Tournament selection with replacement is adopted due to its robustness in multimodal search landscapes and its ability to maintain a balance between exploration and exploitation.

#### 4.6.2. Crossover

To preserve permutation validity, ordered crossover (OX) is employed. Two crossover indices are randomly selected, the corresponding subsequence is inherited from the first parent, and the remaining genes are inserted according to their relative order in the second parent. This operator preserves high-quality subsequences while preventing duplicate neighbourhood assignments [18].

#### 4.6.3. Mutation

To enhance diversity and mitigate premature convergence, adaptive swap mutation is applied. The mutation probability is adjusted according to recent fitness diversity, increasing when the population begins to stagnate and decreasing when sufficient exploration is observed.

#### 4.6.4. Elitism

To preserve the best candidate routes across generations, the top elite chromosomes are directly copied into the subsequent population without modification.

### 4.7. Feasibility Repair Mechanisms

Following crossover and mutation, a repair mechanism is applied to ensure chromosome validity and route feasibility. First, duplicated genes are removed to preserve the permutation constraint. Next, any missing sensing neighbourhoods are reinserted using a greedy cost-aware selection strategy. Candidate sequences that lead to missed sensing neighbourhoods or repeated RRT-Connect failures are then locally adjusted by modifying the visitation order around the infeasible transition. This repair stage improves the likelihood that each chromosome corresponds to a valid 3D-TSPN-compliant sensing route that can be evaluated by the geometric planner.

### 4.8. RRT-Connect for Collision-Free Connectivity

For each consecutive pair of sensing neighbourhoods $\left(N_{i},N_{j}\right)$ in the GA-optimised sequence, a bidirectional RRT-Connect planner is executed to generate a geometrically feasible and obstacle-aware three-dimensional transition path [12,13].

To provide conservative safety margins, each obstacle $O_{k}$ is inflated as follows:

(98) $$\begin{array}{c} O_{k}^{+}=O_{k}⊕B\left(d_{\mathrm{safe}}\right), \end{array}$$

where ⊕ denotes the Minkowski sum, $d_{\mathrm{safe}}$ is the safety margin, and $B(d_{\mathrm{safe}})$ is a ball of radius $d_{\mathrm{safe}}$.

The RRT-Connect planner employs a dual-tree expansion strategy in which one tree is extended toward a randomly sampled point, while the opposite tree attempts to connect to the newly generated node. Specifically, the *Extend* operation incrementally grows the active tree toward the sample, whereas the *Connect* operation advances the opposite tree until a connection is achieved or an obstacle constraint is encountered.

This bidirectional expansion accelerates path discovery in cluttered three-dimensional disaster environments compared with single-tree sampling methods and provides a practical feasibility check for the GA-generated sensing order.

Once a feasible path is obtained, post-processing is applied to improve flight efficiency and numerical consistency [41,42,43]. Specifically, redundant waypoint reduction and uniform path resampling are performed to remove unnecessary intermediate points and produce smoother UAV motion during execution.

### 4.9. Adaptive Minimum-Cost Path Recovery

When the AoI-aware GA and the associated RRT-Connect transitions do not produce a route satisfying all coverage, obstacle, boundary, and path-validity constraints, an adaptive minimum-cost recovery procedure is activated. The recovery module generates multiple coverage-preserving candidate routes, including segmented RRT repair, cluster-aware routing, local repair with coverage restoration, selective high-altitude bridging, and an optimised ceiling-based route. Each candidate is revalidated against the sensing-neighbourhood coverage requirements, inflated obstacle regions, spatial boundaries, and mission-level kinematic and path constraints. Among the feasible candidates, the recovery module selects the route that minimises a weighted cost combining path length, estimated energy consumption, information freshness, and vertical motion. The selected route is then returned to the main mission workflow for safety auditing and execution.

The adaptive minimum-cost recovery mechanism is applied during the pre-mission planning and path-validation stages. It repairs or replaces a route that is found to be infeasible with respect to the obstacles, map boundaries, sensing-neighbourhood coverage, or path-validity constraints already represented in the planning environment before mission execution begins. The current implementation does not perform online trajectory replanning in response to a previously unknown, newly detected, or moving obstacle during flight. During the simulated mission, the active safety layer can apply bounded temporal holds, speed adjustments, or altitude modifications to resolve predicted inter-UAV separation conflicts, but these actions should not be interpreted as general online obstacle replanning. This distinction is a limitation of the present framework. Future work will extend the recovery module with real-time obstacle detection, dynamic map updating, and receding-horizon or incremental online replanning so that the UAVs can respond to newly appearing and moving obstacles while preserving sensing coverage, energy feasibility, information freshness, and inter-UAV safety.

### 4.10. Multi-UAV Conflict Detection and Resolution

To ensure safe simultaneous operation of multiple UAVs, inter-UAV separation is continuously monitored throughout mission execution. For any pair of UAVs *i* and *j*, the instantaneous separation distance is defined as

(99) $$\begin{array}{c} d_{ij}\left(t\right)=∥p_{i}\left(t\right)-p_{j}\left(t\right)∥. \end{array}$$

A potential conflict is detected whenever the separation distance violates the minimum safety threshold:

(100) $$\begin{array}{c} d_{ij}\left(t\right)<d_{\min}. \end{array}$$

#### 4.10.1. Closest-Approach Computation

To reduce the risk of missed conflicts caused by discrete-time sampling, the minimum continuous-time closest-approach distance is evaluated over overlapping trajectory segments:

(101) $$\begin{array}{c} d_{ij}^{\min}=\min_{t\in [t_{s},t_{e}]}d_{ij}\left(t\right), \end{array}$$

where $\left[t_{s},t_{e}\right]$ denotes the temporal overlap interval between the two UAV trajectory segments. Whenever $d_{ij}^{\min}<d_{\min}$, the conflict is recorded and the safety module evaluates a mitigation action.

#### 4.10.2. Resolution Actions

Fixed UAV start-time offsets are applied before mission execution as a preventive temporal-desynchronisation measure. During execution, the continuous-time conflict detector independently evaluates the closest-approach distance between all overlapping UAV trajectory segments. If an unsafe encounter remains or arises, the active resolution module applies a bounded temporal hold, speed adjustment, or spatial altitude modification as a corrective action. The final safety audit then verifies the resulting all-pair separation but does not itself modify the trajectories.

This lightweight resolution strategy preserves the 3D-TSPN and GA-generated sensing order while supporting safe concurrent multi-UAV operation in continuous three-dimensional space.

Overall, the proposed layered architecture unifies global sequencing through AoI-aware GA, coverage flexibility through 3D-TSPN, obstacle-aware geometric feasibility through RRT-Connect, conditionally queue-stable binary computation control through LBO, and multi-UAV safety through continuous closest-approach conflict monitoring. This structured design establishes a resilient UAV-assisted disaster-response pipeline capable of operating reliably under uncertain and dynamic environmental conditions.

## 5. Simulation Setup

This section describes the simulation environment used to evaluate the proposed layered multi-UAV disaster-response framework. The simulation was implemented in MATLAB using custom implementations of 3D-TSPN sensing-neighbourhood modelling, AoI-aware GA sequencing, 3D RRT-Connect path feasibility checking, Lyapunov-based binary MEC offloading, and multi-UAV safety monitoring. The complete set of simulation parameters and default values is summarised in Table 2. Unless otherwise stated, each comparison uses the same environment configuration, task characteristics, channel assumptions, and random-seed approach to ensure a consistent evaluation. The simulated environment consists of a bounded three-dimensional disaster region containing clustered ground sensors, cylindrical obstacle structures, four UAVs, and a single centrally located MEC node. The obstacle structures represent tall physical hazards such as towers or damaged infrastructure. To improve safety under modelling uncertainty, obstacle radii are inflated using a fixed safety margin before path planning is performed.

Ground sensors are placed in clustered regions to emulate realistic post-disaster sensing demand, where critical measurements are often concentrated around damaged areas. Each sensor is represented as a 3D sensing neighbourhood. Therefore, a UAV does not need to visit the exact sensor coordinate; it only needs to enter the corresponding sensing radius to collect the data. This directly supports the 3D-TSPN formulation used by the spatial planning layer [7,44].

The wireless channel between each UAV and the MEC node follows a distance-dependent path-loss model. The channel gain is expressed as

(102) $$\begin{array}{c} h_{i}\left(t\right)=\frac{h_{0}}{r_{i}{\left(t\right)}^{\eta_{\mathrm{pl}}}}, \end{array}$$

where $h_{0}$ is the reference channel gain, $r_{i}\left(t\right)$ is the distance between UAV *i* and the MEC node, and $\eta_{\mathrm{pl}}$ is the path-loss exponent. To emulate disaster-induced wireless uncertainty, the available bandwidth, interference level, and link availability are varied during execution [3,11,45].

For a collected task of size $d_{j}$, the uplink transmission rate is modelled as

(103) $$\begin{array}{c} R_{i}\left(t\right)=B_{i}\left(t\right)\log_{2}\left(1+\frac{P_{i}\left(t\right)h_{i}\left(t\right)}{\sigma_{i}^{2}\left(t\right)+I_{i}\left(t\right)}\right), \end{array}$$

where $B_{i}\left(t\right)$ is the available bandwidth, $P_{i}\left(t\right)$ is the UAV transmit power, $\sigma_{i}^{2}\left(t\right)$ is the noise power, and $I_{i}\left(t\right)$ is the interference power. The corresponding transmission delay is

(104) $$\begin{array}{c} T_{ij}^{\mathrm{tx}}\left(t\right)=\frac{d_{j}}{R_{i}\left(t\right)}. \end{array}$$

The energy-related parameters used in this study are simplified and normalised simulation settings intended to support internally consistent comparisons among the proposed framework, ablation variants, and benchmark methods. In particular, the reported flight, communication, and computation energy values do not represent the full propulsion power profile of a specific commercial quadrotor. Instead, the same energy-accounting model and parameter values are applied consistently to all compared methods so that differences in the reported energy consumption arise from their respective path lengths, mission durations, processing decisions, and communication actions.

The reported results are organised into four distinct experimental configurations. Table 3, Table 4, Table 5, Table 6, Table 7, Table 8 and Table 9 correspond to the default illustrative scenario using seed 42 and the parameter values listed in Table 2. The component-removal experiments use a separate fixed ablation realisation. The same random seed, sensor placement, UAV initial conditions, obstacle layout, system parameters, and algorithmic settings are reused for the complete reference configuration and all ablation variants. The ablation results are, therefore, used only for comparisons within that experiment and are not interpreted as repetitions of the default seed-42 scenario.

Table 10, Table 11 and Table 12 report results over 20 independent stochastic seeds using the system parameters in Table 2. Table 13 and Table 14 correspond to separate controlled benchmark experiments in which the proposed framework and the relevant adapted baseline are evaluated under the same benchmark conditions. Results are compared only within their respective experimental configuration.

If the task is offloaded to the MEC node, the MEC-side processing delay is

(105) $$\begin{array}{c} T_{j}^{\mathrm{edge}}=\frac{d_{j}c_{j}}{f^{\mathrm{mec}}}, \end{array}$$

where $d_{j}$ is the data size of task *j*, $c_{j}$ is the number of CPU cycles per bit, and $f^{\mathrm{mec}}$ is the MEC CPU capacity. Therefore, the total offloading delay is

(106) $$\begin{array}{c} T_{ij}^{\mathrm{off}}\left(t\right)=T_{ij}^{\mathrm{tx}}\left(t\right)+T_{j}^{\mathrm{edge}}. \end{array}$$

Local processing is evaluated using the onboard UAV CPU capacity. For local execution, the processing delay is given by

(107) $$\begin{array}{c} T_{ij}^{\mathrm{loc}}=\frac{d_{j}c_{j}}{f_{i}^{\mathrm{uav}}}, \end{array}$$

where $f_{i}^{\mathrm{uav}}$ is the CPU capacity of UAV *i*. Each collected task is then assigned to either local processing or MEC offloading by the Lyapunov-based binary decision layer, subject to deadline and battery constraints.

The analytical queue model is expressed in a generic slotted form. In the event-driven MATLAB implementation, a decision epoch occurs when a sensor task is collected. One task is added at the corresponding event, and the effective service over the elapsed interval $\delta_{i}\left(t\right)$ is evaluated as ${\tilde{\mu }}_{i}\left(t\right)=\mu_{i}\left(t\right)\delta_{i}\left(t\right)$. Accordingly, the implemented update is

(108) $$Q_{i}\left(t+1\right)=\max\left[Q_{i}\left(t\right)-{\tilde{\mu }}_{i}\left(t\right),0\right]+A_{i}\left(t\right),$$

which has the same queue-update structure used in the Lyapunov derivation. The implementation also imposes a strictly positive lower bound on the candidate processing time before computing the service rate, thereby keeping the effective service finite.

After path generation, the resulting RRT-Connect trajectories are post-processed using redundant waypoint reduction and uniform resampling. This improves path consistency and ensures that the generated UAV trajectories can be evaluated under continuous-time collision monitoring. In the implemented simulation, uniform path resampling is performed using a spatial interval associated with the prescribed UAV reference speed of 1 m/s. Mission timestamps are subsequently constructed from the Euclidean length of each consecutive three-dimensional path segment divided by this reference speed. Consequently, the simulated trajectory represents constant-speed mission-level reference motion. The implementation explicitly verifies spatial boundaries, inflated-obstacle avoidance, sensing-neighbourhood coverage, and continuous-time inter-UAV separation, but it does not explicitly propagate the quadrotor attitude, angular velocity, rotor thrust, or motor dynamics. These lower-level states are assumed to be regulated by an underlying flight controller.

After waypoint reduction and uniform resampling, each post-processed trajectory is revalidated against the inflated obstacle regions and inter-UAV safety constraints. Any trajectory that violates these constraints is rejected or repaired before mission execution. The generated paths are interpreted as mission-level position-reference trajectories for the quadrotor model introduced in Section 2.2. Path post-processing removes redundant waypoints and produces uniformly sampled position references for the prescribed mission-level reference speed. The implemented planner explicitly enforces the stated spatial and translational mission-level constraints, including map boundaries, sensing-neighbourhood coverage, inflated-obstacle clearance, reference speed, and continuous-time inter-UAV separation. Acceleration, attitude, angular-rate, actuator-saturation, and motor-level dynamic feasibility are not explicitly simulated in the present implementation and are assumed to be handled by an underlying low-level flight-control system.

### Monte Carlo Robustness Evaluation

To evaluate the robustness of the proposed layered framework, 20 independent random seeds were considered. For each seed, three processing-control scenarios were executed: PROPOSED, ALL_LOCAL, and ALL_MEC, resulting in 60 total scenario executions. Within each seed, the same stochastic realisation was reused across the three scenarios to enable a paired comparison. All runs used identical system parameters, algorithmic settings, and computational budgets; only the stochastic realisations associated with the random seed were varied. For each seed, the complete spatial, temporal, and safety pipeline was executed, including balanced zone assignment, AoI-aware GA sequencing, 3D-TSPN coverage validation, RRT-Connect path generation, adaptive minimum-cost path recovery when required, Lyapunov-based binary processing control, and continuous-time inter-UAV safety auditing.

For the Monte Carlo analysis, completion-time AoI denotes the elapsed time between the generation or availability of a sensor update and the completion of its selected local or MEC processing mode.

All continuous performance metrics obtained from the 20 independent random seeds are reported as the sample mean and sample standard deviation. For a metric *x*, the 95% confidence interval of the single-policy mean was calculated using the Student’s *t*-distribution as $\bar{x}\pm t_{0.975,n-1}s/\sqrt{n}$, where n=20, $\bar{x}$ is the sample mean, and *s* is the sample standard deviation. Processing-policy comparisons were conducted using two-sided paired-samples *t*-tests because the PROPOSED, ALL_LOCAL, and ALL_MEC policies were evaluated using the same stochastic realisation within each seed, including identical sensing layouts, UAV trajectories, task loads, channel randomness, and mission parameters. For each comparison, the paired difference was defined as the result obtained by the first-listed policy minus that obtained by the reference policy. The 95% confidence interval of the paired difference was calculated as $\bar{d}\pm t_{0.975,n-1}s_{d}/\sqrt{n}$, where $\bar{d}$ and $s_{d}$ are the mean and standard deviation of the 20 within-seed differences, respectively. Statistical significance was assessed using a two-sided significance level of α=0.05, and exact *p*-values are reported. Relative to the all-local policy, the proposed controller reduced total energy consumption from 262.121±10.760 J to 257.204±10.618 J, corresponding to a paired mean difference of −4.917 J (95% CI: [−7.033,−2.800] J; $p=1.082\times 10^{-4}$). It also reduced completion-time AoI from 142.787±17.723 s to 142.694±17.740 s, with a paired mean difference of −0.093 s (95% CI: [−0.138,−0.048] s; $p=3.995\times 10^{-4}$). Relative to the proposed controller, the all-MEC policy increased completion-time AoI by 3.186 s (95% CI: [2.755,3.617] s; $p=3.21\times 10^{-12}$).

An ablation study was conducted to evaluate the individual contribution of the main components of the proposed framework. The study uses the separate fixed ablation realisation defined above. The same random seed, sensor placement, UAV initial states, obstacle layout, and system parameters are maintained across the complete reference configuration and all component-removal variants. Therefore, the ablation reference values are intended only for controlled within-study comparisons and are reported separately from the default seed-42 and benchmark configurations.

The first ablation experiment disabled the balanced zone-assignment module and replaced it with random UAV assignment. As a result, the sensor distribution became unbalanced, with the four UAVs assigned 4, 4, 4, and 8 sensors, respectively. This imbalance increased the system cost from 29.98 to 43.78 and increased the average delay from 13.58 s to 17.58 s. Moreover, the Jain fairness index decreased from 0.9941 to 0.9102, while the weighted fairness index decreased from 0.9523 to 0.9308. These results demonstrate that the balanced clustering module plays an important role in distributing the sensing workload among UAVs, improving fairness, reducing delay, and maintaining a lower overall system cost.

When the GA-based sequencing module was disabled, the framework used a nearest-neighbour sequence instead of evolutionary route optimisation. Although the balanced zone assignment was maintained, the system cost increased substantially from 29.98 to 192.68, while the average delay increased from 13.58 s to 22.62 s. In addition, three sensing tasks were dropped, reducing the number of successfully processed tasks from 20 to 17. The Jain fairness index also decreased from 0.9941 to 0.9265. These results indicate that the GA module is essential for finding efficient visiting sequences and maintaining high sensing coverage under the proposed 3D path-planning constraints.

The removal of the sensing-radius collection mechanism produced the most severe degradation among the ablation variants. In this configuration, UAVs were required to reach the exact sensor locations rather than collecting data within a TSPN-style sensing neighbourhood. This caused repeated GA failures and forced the use of robust fallback paths for all UAVs. Consequently, the system cost increased sharply from 29.98 to 627.79, and the average delay increased from 13.58 s to 52.57 s. Furthermore, only eight tasks were processed successfully, while twelve tasks were dropped. The Jain fairness index decreased from 0.9941 to 0.6154, confirming that sensing-radius collection is a critical component for improving feasibility, coverage, delay, and workload balance in obstacle-constrained 3D environments.

To assess the role of Lyapunov-based processing control, the dynamic local/MEC decision module was disabled and all collected tasks were processed locally. Under this fixed and relatively favourable scenario, the all-local configuration achieved a slightly lower system cost of 29.48 compared with 29.98 for the complete framework, and reduced the average delay from 13.58 s to 10.26 s. This result shows that MEC offloading is not necessarily beneficial for every task or operating condition. When the workload is light, the onboard CPU is sufficient, and local processing satisfies the deadline and energy constraints so that local-only execution can be competitive.

However, the all-local policy increased the average energy consumption per task from 4.47 J to 4.85 J and reduced the weighted fairness index from 0.9523 to 0.8773. In contrast, the complete controller retained MEC as an adaptive option and selected it only when the corresponding drift-plus-penalty objective was lower than that of local processing. These results indicate that the contribution of the MEC component is not to dominate local processing in every individual scenario, but to provide an additional feasible processing mode that can improve the balance among energy consumption, completion-time AoI, queue behaviour, and resource utilisation when local execution becomes comparatively costly or constrained.

The safety-avoidance module was also disabled to evaluate its contribution to robust multi-UAV operation. Without this module, the system cost increased from 29.98 to 32.44, while the weighted fairness index decreased from 0.9523 to 0.9121. Although this configuration still processed all 20 tasks and maintained a high Jain fairness index of 0.9899, removing safety-aware avoidance reduces the framework’s ability to proactively regulate UAV interactions during simultaneous missions. Therefore, the safety module contributes to maintaining a more reliable and balanced mission execution, particularly in shared airspace where near-miss and buffer-intrusion risks must be controlled.

Finally, the RRT-Connect obstacle-aware planner was disabled and replaced with direct path segments without tower inflation. This resulted in a substantial increase in system cost, from 29.98 to 593.21. Although the average delay remained comparable at 13.03 s, and all 20 tasks were processed, the weighted fairness index dropped significantly, from 0.9523 to 0.7486. This indicates that direct routing may appear efficient in terms of travel time, but it fails to provide the same level of obstacle-aware feasibility and balanced mission quality. Therefore, the RRT-Connect component is essential for maintaining safe and realistic 3D path generation in obstacle-constrained environments. For clarity, mission completion time is defined as the spatial mission makespan

(109) $$T^{\mathrm{mission}}=\max_{i\in U}t_{i}^{\mathrm{end}},$$

where $t_{i}^{\mathrm{end}}$ is the final execution time of UAV *i*’s validated mission trajectory after accounting for its start-time offset and any temporal adjustment introduced by the safety-resolution mechanism. Mission completion time is reported separately from per-task processing delay and completion-time AoI. Overall, these ablation results indicate that each component contributes meaningfully to the proposed framework, as removing any individual module leads to degradation in at least one key performance aspect, including cost, delay, coverage, fairness, energy efficiency, or operational robustness.

## 6. Experimental Evaluation and Results’ Discussion

This section evaluates the proposed 3D-TSPN–AoI-GA–RRT-Connect–LBO framework under the simulation setting described above. The objective is to quantify the contribution of layered spatial planning, Lyapunov-based binary MEC offloading, and multi-UAV safety monitoring to mission success, timeliness, energy efficiency, queue stability, and operational safety.

The evaluation is organised into three parts: comparative performance against representative baseline planners, single-UAV versus multi-UAV scalability, and system-level robustness in terms of processing decisions, queue stability, and safety.

### 6.1. Comparative Benchmarking Against Baseline Planners

Table 3 compares the proposed framework against representative baseline configurations. The baselines are designed to isolate the contribution of the main retained components: 3D-TSPN coverage modelling, AoI-aware GA sequencing, RRT-Connect path feasibility checking, and Lyapunov-based binary MEC offloading.

Table 3 compares the proposed full framework with a nearest-neighbour multi-UAV baseline and a single-UAV baseline. The proposed framework achieved complete sensing coverage with a 100% success rate, while maintaining zero obstacle collisions and zero inter-UAV collisions. In contrast, the nearest-neighbour baseline achieved only 85% coverage and produced 17 obstacle collisions. This demonstrates that simple greedy routing is insufficient in obstacle-constrained disaster-response environments, where route feasibility and safety-aware planning are required [23,44].

Compared with the nearest-neighbour baseline, the proposed framework reduced the average AoI from 145.76 s to 74.24 s, corresponding to a 49.1% reduction. It also reduced the total energy consumption from 325.81 J to 266.96 J and the mission completion time from 293.80 s to 210.66 s, corresponding to reductions of 18.1% and 28.3%, respectively. These improvements are achieved because the proposed framework combines sensing-neighbourhood planning, AoI-aware routing, obstacle-aware RRT-Connect feasibility checking, and final safety monitoring, rather than relying only on the closest next sensor.

### 6.2. Impact of Multi-UAV Coordination

To quantify the importance of cooperative deployment, the proposed framework is evaluated in a single-UAV setting and compared against the default four-UAV configuration. This comparison distinguishes apparent efficiency from true mission effectiveness [25,38,46].

Table 4 shows the benefit of using a coordinated multi-UAV framework instead of a single UAV. Both methods achieved 100% coverage; however, the proposed four-UAV framework reduced the collection time from 460.92 s to 210.66 s, corresponding to a 54.3% reduction. The average AoI was also reduced from 151.56 s to 74.24 s, corresponding to a 51.0% reduction. These results indicate that distributing the sensing workload across multiple UAVs significantly improves mission efficiency and information freshness.

The proposed framework consumed more energy than the single-UAV baseline because four UAVs were deployed instead of one. Specifically, the total energy increased from 189.09 J to 266.96 J. However, this moderate increase in energy consumption is associated with substantial reductions in mission completion time and AoI. Therefore, the proposed framework provides a favourable trade-off for disaster-response scenarios where timely data collection and information freshness are more critical than minimising the total energy of a single vehicle.

### 6.3. Processing Decisions and Queue Stability

The Lyapunov-based temporal layer determines whether each collected task is processed locally or offloaded to the MEC node. Table 5 summarises the binary processing decisions and final queue backlogs of the proposed framework. The Lyapunov-based temporal layer selected local processing for 19 tasks and MEC offloading for 1 task, with no dropped tasks. This indicates that most collected tasks were locally feasible under the considered energy, delay, and channel conditions, while MEC offloading was selected only when it reduced the Lyapunov drift-plus-penalty cost. The final queue backlogs remained bounded across all UAVs, ranging from 4.49 to 7.12 tasks, which provides finite-horizon empirical evidence of bounded queue behaviour during the simulated mission.

The Lyapunov control parameter was set to V=8 in all reported experiments. This value was used as a nominal setting to balance the queue-reduction term and the weighted energy–delay–AoI penalty in the drift-plus-penalty objective. It is not claimed to be uniquely optimal or universally suitable for all operating conditions. A systematic sensitivity analysis over multiple values of *V* was not conducted in the present study. Such an analysis under different workloads, queue states, wireless conditions, and penalty weights will be considered in future work.

The corresponding collision and separation results are analysed separately in Section 6.4.

Overall, the results demonstrate that the proposed layered framework provides the most balanced performance across coverage, AoI, mission time, energy consumption, and safety. The nearest-neighbour baseline is less reliable because it fails to achieve full coverage and results in obstacle collisions. The single-UAV baseline is safe and energy-efficient, but it suffers from substantially longer mission completion time and higher AoI. By combining 3D-TSPN sensing neighbourhoods, AoI-aware GA routing, RRT-Connect path feasibility, Lyapunov-based binary processing, and safety monitoring, the proposed framework achieves complete coverage, lower AoI, shorter mission time, bounded queues, and collision-free execution. MEC offloading was selected selectively rather than continuously, reflecting the queue-aware and channel-aware nature of the Lyapunov controller.

The distribution of LOCAL and MEC decisions reflects the interaction among queue backlog, remaining UAV energy, task size, processing deadlines, onboard CPU capability, UAV–MEC distance, and stochastic wireless-channel quality. Local processing is generally preferred when the onboard CPU can complete the task within its deadline with acceptable energy consumption. MEC offloading becomes preferable only when the wireless link is sufficiently strong, the combined transmission and edge-processing delay remains feasible, and the resulting drift-plus-penalty objective is lower than that of local execution.

Accordingly, a low MEC-use rate does not indicate that the controller is inactive or ineffective. It indicates that local processing is the lower-cost feasible action for most tasks under the evaluated scenario. The value of the MEC option becomes clearer under task and channel realisations in which local computation is relatively energy-intensive or slow, queue pressure is higher, or a favourable wireless link enables efficient edge processing. A task is dropped only when neither local processing nor MEC offloading satisfies the current deadline, energy, and communication-feasibility constraints.

Dropped tasks occur only when neither local processing nor MEC offloading is feasible under the current energy, deadline, and channel conditions. Therefore, the number of dropped tasks provides an important indicator of whether the temporal layer can sustain reliable computation under disaster-induced communication uncertainty.

### 6.4. Safety and Collision Analysis

Operational safety is evaluated using obstacle collision counts, inter-UAV collision counts, and buffer-zone intrusions.

Table 7 shows the safety and collision-monitoring outcomes of the proposed framework. The proposed method achieved zero real obstacle collisions and zero inter-UAV collisions. In addition, no near misses or buffer-zone intrusions were detected. These results indicate that the combination of RRT-Connect feasibility checking, inflated obstacle modelling, final path-safety repair, and temporal deconfliction successfully maintained safe multi-UAV operation in the 3D disaster-response environment. The RRT-Connect planner, inflated obstacle representation, and continuous-time collision monitoring jointly support safe operation in the 3D environment. Real obstacle collisions indicate direct violations of physical obstacles, while buffer-zone intrusions indicate that the UAV entered the conservative safety margin around an obstacle. Inter-UAV collisions and near misses provide insight into the effectiveness of temporal desynchronisation and speed-adjustment-based mitigation.

A desirable outcome is, therefore, not only a high collection success rate, but also zero real obstacle collisions and no inter-UAV collisions. Buffer-zone intrusions, if present, should be interpreted as conservative safety warnings rather than physical impacts.

#### Start-Offset and Conflict-Resolution Ablation

To separate the preventive effect of fixed temporal desynchronisation from the corrective effect of active conflict resolution, a controlled single-seed 2×2 safety ablation was conducted. The same random seed, sensor and obstacle realisation, planned UAV paths, motion parameters, minimum-separation threshold, collision threshold, and safety-audit time step were retained across all four configurations. Only the fixed start-offset and active resolution switches were varied. The experiment was intended to isolate the functional contribution of the two safety mechanisms rather than provide a population-level statistical comparison. This controlled ablation demonstrates functional causality in the evaluated realisation, while the 20-seed complete-framework results provide complementary evidence of safety robustness.

Without start offsets or active resolution, one unsafe UAV pair remained unresolved and produced a final collision event, with the minimum separation decreasing to 1.095 m. Consequently, the corresponding mission was classified as invalid. Enabling fixed start offsets alone eliminated the initial unsafe encounter and increased the minimum separation to 11.015 m without requiring any active resolution action. This demonstrates the preventive role of temporal desynchronisation. However, the offsets increased the mission completion time from 207.01 s to 268.62 s.

When active conflict resolution was enabled without fixed offsets, the same initial unsafe pair was detected, but a single corrective action removed the final collision and near-miss events. The resulting minimum separation was 10.079 m, which exceeded the required 10 m safety threshold, and the mission remained valid. The mission completion time was 214.12 s, only 7.11 s greater than the unconstrained case and substantially lower than that of the fixed-offset configuration. Therefore, in this controlled realisation, the active mechanism independently restored safe separation, with a smaller mission-time penalty than fixed temporal offsets.

When both mechanisms were enabled, the fixed offsets prevented the unsafe encounter before active intervention was required, and the resolution-action count remained zero. These results distinguish the roles of the two mechanisms: fixed offsets provide preventive temporal desynchronisation, whereas active conflict resolution provides an adaptive corrective capability when unsafe encounters remain. Their combined use, therefore, creates complementary preventive and corrective safety layers. Although the factorial ablation is reported for one controlled realisation to isolate the two mechanisms, the complete configuration also produced zero final collision and near-miss events across all 20 independent random-seed runs, supporting the robustness of the combined preventive and corrective safety design.

### 6.5. Discussion

Overall, the proposed framework achieves balanced performance because the spatial, temporal, and safety layers address complementary mission requirements. The spatial layer determines where UAVs should fly and the order in which sensing neighbourhoods should be visited. The temporal layer determines how collected tasks should be processed under queue, energy, delay, AoI, and channel constraints. The safety layer monitors whether simultaneous UAV execution remains collision-free.

The main scientific insight is that disaster-response performance cannot be evaluated using travel distance or energy consumption alone. A route that is short may still produce stale information, unsafe UAV interactions, or infeasible computation decisions. Similarly, an offloading strategy that reduces local computation may fail under weak wireless conditions. The proposed layered design, therefore, provides a practical balance between sensing coverage, freshness, computation feasibility, and operational safety.

### 6.6. System-Level Behaviour and Safety

In addition to mission-level outcomes, system robustness is evaluated through coverage success, processing feasibility, queue stability, energy consumption, AoI, and collision-related metrics. These metrics provide a broader view of whether the proposed framework can operate reliably under obstacle constraints, stochastic wireless conditions, and concurrent multi-UAV execution.

Table 9 summarises the system-level robustness of the proposed framework. The framework achieved complete sensing coverage with a 100% success rate, while maintaining an average AoI of 74.24 s and a total collection time of 210.66 s. The total mission-energy consumption was 266.96 J. The Lyapunov-based temporal layer selected local processing for 19 tasks and MEC offloading for 1 task, with no dropped tasks. The final queue backlogs remained bounded across all UAVs, ranging from 4.49 to 7.12 tasks.

The default-scenario safety outcomes are summarised in Table 9 and analysed in detail in Section 6.4.

The processing decision statistics provide insight into the behaviour of the Lyapunov-based temporal layer. LOCAL decisions indicate tasks processed onboard the UAV, whereas MEC decisions indicate tasks offloaded to the edge node. Dropped tasks occur only when neither local processing nor MEC offloading satisfies the current energy, delay, and channel feasibility constraints. Therefore, a low dropped-task count indicates that the temporal layer can maintain useful service under stochastic communication conditions.

The waypoint reduction and path resampling stages also contribute to execution consistency by removing unnecessary intermediate points and generating more regular UAV movement. However, these post-processing steps are used to improve path representation and simulation stability rather than to replace the obstacle-aware feasibility role of RRT-Connect.

### 6.7. Robustness Across 20 Independent Random Seeds

The Monte Carlo statistics reported in this subsection correspond to the complete stochastic mission evaluation and should not be interpreted as replacements for the fixed-scenario baseline or controlled benchmark results reported in the preceding subsections. The latter are retained for paired method comparison under identical individual scenarios, whereas the present experiment quantifies run-to-run robustness across independent stochastic realisations.

Table 10 and Table 11 summarise the robustness evaluation across 20 independent stochastic realisations. The proposed framework completed all runs successfully, achieved complete sensing coverage in every run, and produced no dropped tasks. After conflict resolution and final trajectory validation, no collision or near-miss events remained, and every run passed the all-pair continuous-time safety audit. The smallest separation observed across all UAV pairs and seeds was 10.002 m, which remained above the prescribed 10 m minimum-separation threshold.

The mean completion-time AoI was 142.694 s, with a standard deviation of 17.740 s and a 95% confidence interval of [134.391,150.997] s. The mean mission completion time was 314.098 s, with a standard deviation of 39.875 s and a 95% confidence interval of [295.436,332.760] s. The mean total energy consumption was 257.204 J, with a standard deviation of 10.618 J and a 95% confidence interval of [252.235,262.174] J. These intervals quantify the run-to-run variability introduced by stochastic route generation, channel conditions, task processing states, and conflict-resolution actions. The adaptive minimum-cost recovery mechanism was activated in only one of the 20 runs. In that realisation, two candidate recovery routes satisfied the final feasibility checks, and the module selected the LOCAL_REPAIR_PLUS_COVERAGE solution. The selected recovery trajectory had a length of 298.183 m and preserved complete sensing coverage, obstacle feasibility, and inter-UAV safety. The limited activation frequency indicates that the nominal planner was feasible in most stochastic realisations, while the successful isolated recovery case demonstrates that the framework can retain mission validity under a difficult planning outcome. The recorded queue states remained finite during the evaluated mission horizons. However, these finite-duration observations should be interpreted only as empirical evidence of queue behaviour under the implemented controller and not as an independent proof of infinite-horizon strong queue stability. The analytical stability result remains conditional on the assumptions of Theorem 1, including arrival rates within the stability region and the existence of a feasible stabilising policy.

#### Paired Processing-Policy Comparison

To isolate the effect of the processing policy, each of the 20 random seeds was evaluated under three processing-control configurations: the proposed Lyapunov controller, an all-local policy, and an all-MEC policy. Within each seed, all three configurations used the same sensing realisation, UAV trajectories, task loads, channel randomness, and mission parameters. This paired design ensures that the observed differences in completion-time AoI and energy consumption arise from the processing policy rather than from different spatial or wireless realisations.

Under the all-local policy, every collected task is processed onboard the UAV. Under the all-MEC policy, MEC offloading is selected whenever the MEC mode satisfies the wireless-link, deadline, and energy feasibility constraints; local processing is retained as a fallback when MEC offloading is infeasible. Consequently, the all-MEC policy should be interpreted as an MEC-preferred feasible policy rather than as forced offloading of every collected task.

As shown in Table 12, the proposed controller used MEC selectively, with a mean MEC-use rate of only 5.25%, and selected at least one MEC task in 13 of the 20 runs. Relative to the all-local policy, the proposed controller reduced mean total energy consumption from 262.121 J to 257.204 J. The paired mean difference, calculated as proposed minus all-local, was −4.917 J, with a 95% confidence interval of [−7.033,−2.800] J and $p=1.082\times 10^{-4}$. The energy reduction occurred in every run in which the proposed controller selected at least one MEC task, whereas runs with no MEC selection reproduced the all-local outcome. This result shows that the controller activates MEC selectively when the evaluated task, queue, energy, and channel conditions make offloading beneficial.

The proposed controller also reduced mean completion-time AoI from 142.787 s under all-local processing to 142.694 s. The paired mean difference was −0.093 s, with a 95% confidence interval of [−0.138,−0.048] s and $p=3.995\times 10^{-4}$. Although this freshness improvement is small in magnitude, it is statistically significant and is reported without claiming a large AoI advantage over all-local execution. Mission completion time remained unchanged because the three processing policies used identical UAV trajectories and the processing decisions did not alter the spatial mission schedule.

The all-MEC policy achieved the lowest mean energy consumption, 249.524 J, but increased mean completion-time AoI to 145.880 s. Relative to the proposed controller, the all-MEC policy increased completion-time AoI by 3.186 s, with a paired 95% confidence interval of [2.755,3.617] s and $p=3.21\times 10^{-12}$. This result indicates that more aggressive MEC use can provide additional energy savings but may reduce information freshness because of the associated transmission and edge-processing delays.

The proposed controller, therefore, provides an intermediate and adaptive operating point. It uses MEC selectively to capture energy-saving opportunities while avoiding the completion-time AoI degradation associated with excessive offloading. These results confirm that the benefit of MEC is conditional rather than universal. MEC is most useful when favourable channel conditions, local processing limitations, queue state, task requirements, or local energy costs make offloading the lower-cost feasible action. Under light workloads and favourable onboard-computation conditions, the controller may correctly select few or no MEC actions and behave similarly to the all-local policy.

### 6.8. MEC-Offloading Sensitivity Analysis

To examine whether the low MEC utilisation observed under the baseline configuration was caused by an inherent limitation of the Lyapunov controller or by the evaluated operating conditions, a sensitivity experiment was conducted under three processing regimes using the same 20 matched random seeds in each regime. The local-processing-favourable condition combined stronger onboard computation with less favourable wireless conditions, the balanced condition provided comparable local and MEC capabilities, and the MEC-favourable condition combined slower and more energy-intensive local processing with stronger wireless connectivity and higher MEC capacity. The results demonstrate a clear adaptation of the processing decisions to the operating environment.

Table 14 summarises the MEC-offloading sensitivity experiment across the three operating regimes. The controller shifted from fully local execution under local-processing-favourable conditions to a mixed decision pattern under balanced conditions and then to almost complete MEC offloading under MEC-favourable conditions. Specifically, the mean MEC-use rate increased from 0.00% to 54.00% and then to 98.00%. This transition was accompanied by systematic changes in processing delay, completion-time AoI, energy consumption, and final queue backlog. No tasks were dropped under the local-favourable or balanced conditions. Under MEC-favourable conditions, the mean number of dropped tasks was only 0.35±0.67 per run, corresponding to a small number of tasks for which neither candidate mode remained feasible. These results demonstrate that the low MEC-use rate observed under the baseline configuration was caused by its particular computation, energy, queue, and wireless conditions rather than by a fixed preference for local processing in the Lyapunov controller.

Under local-processing-favourable conditions, the controller selected LOCAL processing for 100.00%±0.00% of the tasks, with a 95% confidence interval of [100.00,100.00]%, while the MEC rate was 0%. Under balanced conditions, the mean LOCAL and MEC decision rates were 46.00%±11.99% and 54.00%±11.99%, with 95% confidence intervals of [40.39,51.61]% and [48.39,59.61]%, respectively. Under MEC-favourable conditions, the MEC rate increased to 98.00%±3.40%, with a 95% confidence interval of [96.41,99.59]%, whereas the mean LOCAL rate decreased to 0.25%±1.12%. The corresponding mean processing delays were 2.716±0.084, 6.786±0.531, and 2.424±0.255 s, while the mean completion-time AoI values were 137.58±17.79, 141.65±17.78, and 134.67±19.13 s for the local-favourable, balanced, and MEC-favourable conditions, respectively. Mean total energy consumption was 186.06±10.80, 238.21±21.43, and 169.36±12.51 J, and the corresponding mean final queue backlogs were 1.108±0.168, 1.432±0.277, and 1.096±0.139. No tasks were dropped in the local-favourable or balanced conditions, whereas the MEC-favourable condition produced 0.35±0.67 dropped tasks per run because a small number of tasks became infeasible despite the high MEC-selection rate. The reported dropped-task count refers only to post-collection processing infeasibility and does not indicate a loss of sensing coverage; all sensor updates were successfully collected, while a small number of the resulting computation tasks could not satisfy the instantaneous LOCAL or MEC feasibility constraints. The 95% confidence intervals were calculated as $\bar{x}\pm t_{0.975,n-1}s/\sqrt{n}$. These results confirm that the previously reported low MEC-use rate reflects the particular computation and communication conditions of the baseline scenario rather than a fixed bias in the controller; the proposed decision mechanism can move from fully local processing to mixed execution and then to almost complete MEC offloading as local CPU capacity, wireless quality, task demand, energy cost, and queue pressure change.

### 6.9. Comparison with Recent Benchmark Methods

The benchmark-comparison experiments were conducted under a controlled benchmark scenario to ensure that the proposed framework and both benchmark methods were evaluated using the same network size, obstacle layout, sensing workload, and simulation configuration. Therefore, the numerical values reported in Table 15 and Table 16 differ from the fixed-scenario internal baseline results reported earlier and from the Monte Carlo robustness results in Table 10 and Table 11. The two benchmark tables report results obtained from separate controlled comparison experiments.

To provide broader validation, the proposed framework is compared against two categories of benchmark methods: planning-oriented multi-UAV optimisation methods and MEC-oriented cooperative offloading methods [47]. Planning-oriented benchmarks mainly focus on task allocation, route sequencing, and mission completion time, while MEC-oriented benchmarks mainly evaluate delay, energy consumption, computation offloading, and fairness.

The proposed framework differs from both categories because it evaluates mission performance through a layered spatial–temporal architecture. The spatial layer combines 3D-TSPN sensing-neighbourhood coverage, AoI-aware GA sequencing, and RRT-Connect path feasibility checking, while the temporal layer uses Lyapunov-based binary local/MEC processing decisions. Therefore, the aim of the comparison is not to claim superiority over specialised routing-only or MEC-only methods in every isolated metric, but to evaluate whether the proposed framework provides a more balanced solution for realistic disaster-response missions involving coverage, freshness, computation feasibility, and safety.

For reproducibility, all benchmark parameters were fixed before the reported evaluation and were not adjusted using the final test results. The modified GA used a population of 200 chromosomes, 200 generations, 10 elite solutions, a crossover probability of 0.90, and assignment and ordering mutation probabilities of 0.08 and 0.15, respectively. The Benchmark 2-adapted cooperative MEC baseline used delay and energy weights of 0.55 and 0.45, a maximum of 12 SCA iterations, and a convergence tolerance of $10^{-4}$.

All methods were evaluated using the same numbers of UAVs and sensors, obstacle layout, sensing workload, initial conditions, energy-accounting model, and metric definitions. Identical iteration counts were not imposed because the methods have different optimisation structures. Instead, algorithm-specific computational budgets were retained and runtime was reported separately to make this distinction transparent.

### 6.10. Comparison with Benchmark 1: A Modified GA Planning Baseline

To provide a fair planning-oriented comparison, the proposed framework was evaluated against a modified GA planning baseline inspired by the work of Yan et al. [47]. The reference method addresses integrated multi-UAV task allocation and path planning using a modified genetic algorithm (GA), where task allocation and route planning are optimised jointly under resource-related and simultaneous-arrival constraints. Since the original benchmark was designed for cooperative multi-UAV attacking missions rather than disaster-response sensing, it was adapted in this work to the proposed UAV-based data-collection environment.

In the adapted baseline, sensor nodes are treated as data-collection tasks, and the UAVs operate in the same 3D environment, with the same number of UAVs, sensors, and cylindrical obstacle structures. The baseline follows the main planning-oriented principles of the reference method by jointly encoding UAV assignment and route ordering within the GA. Specifically, each candidate solution assigns every sensor to one UAV and determines the visiting order for the sensors allocated to each UAV. The objective function combines the total travel distance across all UAVs and the maximum individual UAV route length, with additional penalties for infeasible or unsafe routes. This makes the baseline suitable for evaluating the planning contribution of the proposed framework under the same simulation setting.

The comparison is, therefore, not a direct reuse of numerical values reported in the reference paper. Instead, it is a re-implementation of the core modified GA-planning idea inside the proposed simulation environment. This avoids unfair comparison caused by differences in mission type, region size, UAV dynamics, target/task definitions, and obstacle assumptions. The purpose of the baseline is to determine whether the proposed layered framework provides measurable advantages beyond GA-based task allocation and route sequencing alone.

Several simplifications are retained in the adapted baseline. It does not include 3D-TSPN sensing neighbourhoods, AoI-aware route sequencing, RRT-Connect-based path feasibility checking, Lyapunov-based binary MEC offloading, queue-stability control, or temporal inter-UAV deconfliction. Obstacle handling is implemented through a simplified deterministic feasibility mechanism and post-evaluation safety checking. Therefore, the modified GA baseline should be interpreted as a planning-only benchmark rather than a complete spatial–temporal disaster-response framework.

Table 15 compares the proposed framework with the adapted modified GA planning baseline using the final shared metrics. Both methods achieved complete sensing coverage, collecting all 20 sensors with a 100% success rate. Both methods also avoided actual obstacle collisions, inter-UAV collisions, buffer-zone intrusions, and dropped tasks. However, the proposed framework achieved substantially stronger time-sensitive and energy-aware performance.

The average AoI was reduced from 183.74 s in the modified GA baseline to 83.93 s in the proposed framework, corresponding to a 54.3% reduction. The total collection time was also reduced from 396.29 s to 168.91 s, corresponding to a 57.4% reduction. These improvements demonstrate that the proposed framework provides significantly fresher information and faster mission completion than GA-based task allocation and route sequencing alone.

The proposed framework also reduced total energy consumption from 265.91 J to 244.50 J, corresponding to an 8.1% reduction. Similarly, the average energy per collected sensor decreased from 13.30 J to 12.23 J. This shows that the improvement in AoI and collection time is not achieved at the expense of energy consumption; instead, the proposed framework improves freshness, mission speed, and energy efficiency simultaneously.

The fairness index of the proposed framework was 0.9705, compared with 0.9804 for the modified GA baseline. Although the modified GA baseline achieved slightly higher fairness, the difference is small, and both values indicate a highly balanced multi-UAV workload distribution. Overall, the comparison shows that GA-based task allocation and route sequencing alone are not sufficient for time-critical disaster-response sensing. By integrating 3D-TSPN sensing neighbourhoods, AoI-aware sequencing, RRT-Connect feasibility checking, Lyapunov-based binary processing, and safety monitoring, the proposed framework provides a more complete spatial–temporal solution than the planning-only modified GA baseline.

**Figure 7:**
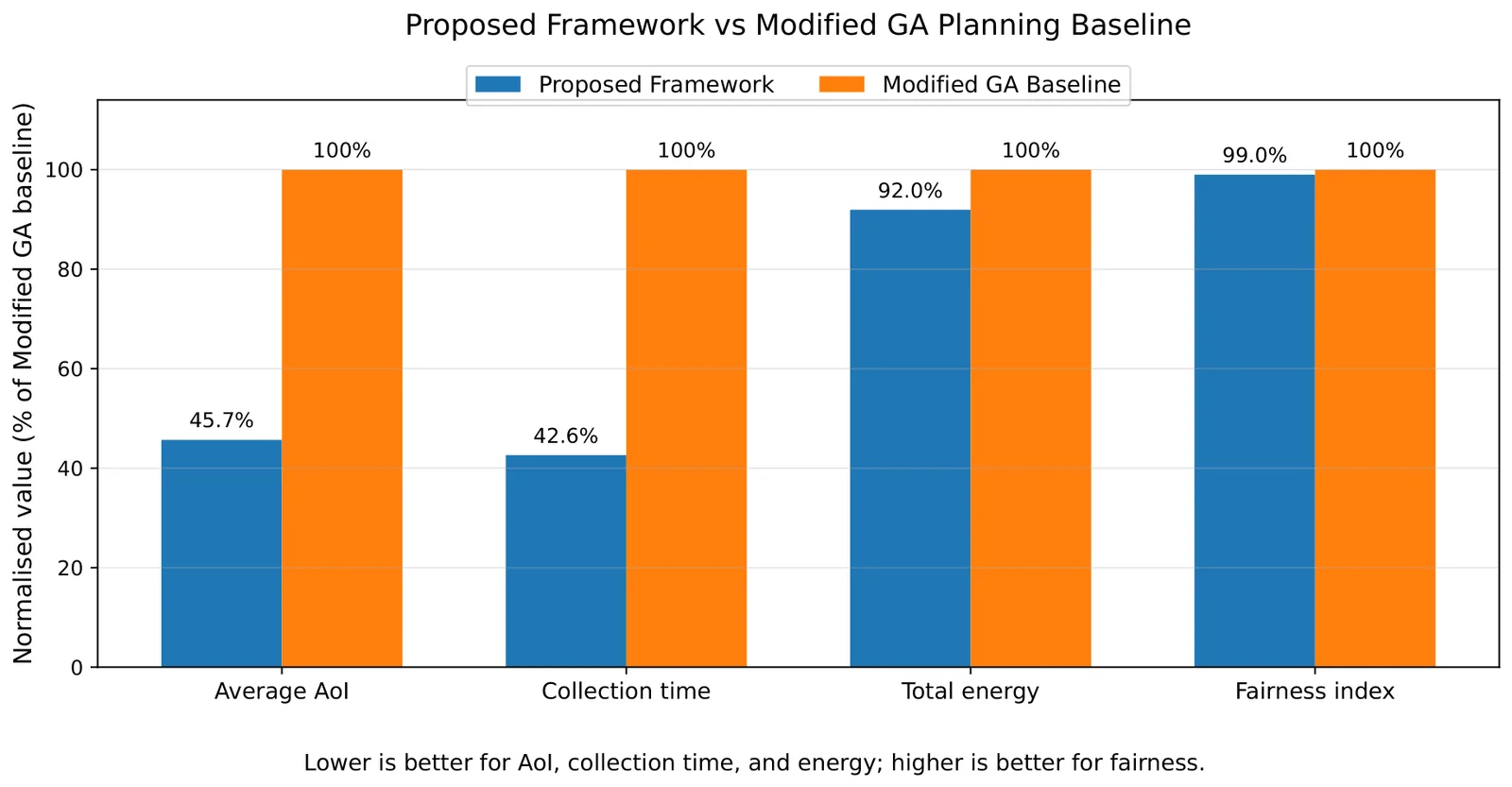
Normalised performance comparison with the modified GA baseline.

Figure 7 presents a benefit-oriented normalised comparison of the AoI, collection-time, energy, and fairness results reported in Table 15. For all four metrics, a larger normalised score indicates better performance.

### 6.11. Comparison with Benchmark 2: Adapted Cooperative MEC Baseline

To further evaluate the effectiveness of the proposed framework, we compare it with an MEC-oriented benchmark adapted from the multi-UAV cooperative computation framework proposed in Benchmark 2 [48]. This benchmark is particularly relevant because it addresses joint trajectory planning and computation resource allocation in a multi-UAV MEC system. In the original study, the optimisation problem is decomposed into two coupled time scales. Over the long time scale, Proximal Policy Optimisation (PPO) is used for multi-UAV trajectory planning under dynamic ground-terminal mobility and random task arrivals. Over the short time scale, greedy association is first used to assign ground terminals to UAVs, after which Successive Convex Approximation (SCA) and CVX are used to optimise the inter-UAV task offloading ratios and UAV computation resource allocation.

This adapted benchmark provides a strong comparison point for the proposed framework because it represents a computation-centric cooperative MEC strategy. Unlike simple routing or nearest-neighbour baselines, Benchmark 2 explicitly considers cooperative processing among UAVs and optimises delay–energy trade-offs through resource allocation. Therefore, comparing against this method helps evaluate whether the proposed framework remains effective against a recent MEC-oriented optimisation approach rather than only against planning-based baselines.

For a fair evaluation, the Benchmark 2-inspired method was adapted to the same disaster-response simulation environment used by the proposed framework, including the same number of UAVs, sensors, sensing region, obstacle layout, initial UAV conditions, sensing workload, energy accounting, and metric definitions.

The adaptation retains the two-timescale organisation of the reference method. Over the long timescale, a PPO-inspired positioning stage represents the UAV-positioning component of the original framework. Over the short timescale, greedy association assigns sensing tasks to UAVs, after which SCA/CVX-based optimisation determines the cooperative computation and resource-allocation decisions. The long-timescale stage is an adapted positioning mechanism and should not be interpreted as a separately trained and validated PPO neural policy.

The purpose of this adaptation is to preserve the computation-oriented structure of the reference method while enabling a controlled comparison under the common disaster-response setting. The implemented baseline, therefore, represents a Benchmark 2-inspired cooperative MEC method rather than an exact reproduction of the original trained PPO system. In contrast, the proposed framework uses a layered spatial–temporal–safety architecture based on 3D-TSPN sensing neighbourhoods, AoI-aware GA sequencing, RRT-Connect path feasibility checking, Lyapunov-based binary local/MEC offloading, and multi-UAV safety monitoring.

This comparison is important because the two methods emphasise different design philosophies. Benchmark 2 focuses primarily on cooperative computation and resource allocation, whereas the proposed framework targets disaster-response data collection where information freshness, obstacle-aware 3D feasibility, queue-stable binary offloading, and safe multi-UAV coordination are jointly required. Therefore, the comparison highlights not only delay, energy, and system cost performance, but also the operational suitability of each method for safety-critical disaster-response missions.

**Figure 8:**
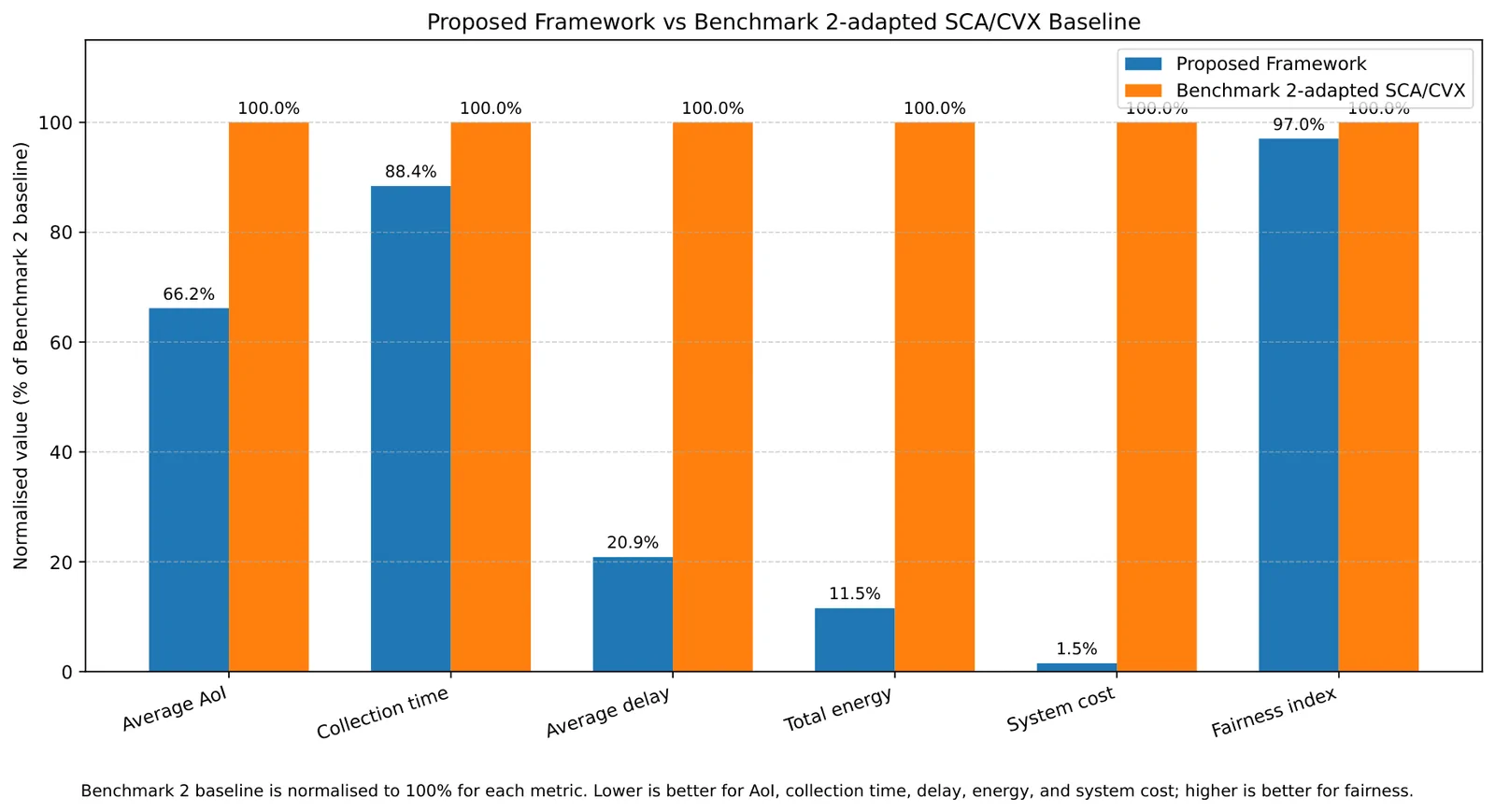
Normalised performance comparison between the proposed framework and the Benchmark 2-adapted cooperative MEC baseline. The benchmark baseline is normalised to 100% for each metric.

Figure 8 shows that the proposed framework outperforms the Benchmark 2-adapted baseline in AoI, delay, energy consumption, and system cost, while maintaining comparable fairness.

Table 16 compares the proposed framework with the Benchmark 2-adapted cooperative MEC baseline under the same number of UAVs and sensors. Both methods achieved complete sensing coverage with a 100% success rate and no dropped tasks. However, the proposed framework achieved stronger overall performance across the main disaster-response metrics.

The proposed framework reduced the total collection time from 191.05 s to 168.91 s, corresponding to an 11.6% improvement. It also reduced the average AoI from 126.84 s to 83.93 s, corresponding to a 33.8% improvement in information freshness. The average delay per collected sensor decreased from 40.49 s to 8.45 s, while the average processing delay decreased from 40.07 s to 7.46 s. In addition, the proposed framework reduced the total energy consumption from 2119.53 J to 244.50 J, corresponding to an 88.5% reduction, and reduced the system cost from 1399.15 to 21.31. The substantially higher energy consumption of the Benchmark 2-adapted baseline is primarily caused by more frequent UAV movement, additional cooperative processing, longer active computation periods, and a different resource-allocation pattern. Both methods were evaluated using the same energy-accounting model and simulation conditions.

From a safety perspective, both methods avoided obstacle collisions and buffer-zone intrusions. Nevertheless, the Benchmark 2-adapted baseline produced 24 inter-UAV collisions and 7 near misses, whereas the proposed framework achieved zero inter-UAV collisions and only 2 near misses. This demonstrates that the proposed spatial–temporal–safety integration provides substantially safer multi-UAV coordination in the evaluated disaster-response environment.

The Benchmark 2-adapted baseline achieved slightly higher fairness values, with a Jain fairness index of 1.0000 compared with 0.9705 for the proposed framework. This is expected because the cooperative SCA/CVX allocation distributes computation load almost uniformly across UAVs. However, this fairness advantage is obtained at the cost of higher delay, higher AoI, significantly higher energy consumption, and severe inter-UAV collision risk.

**Figure 9:**
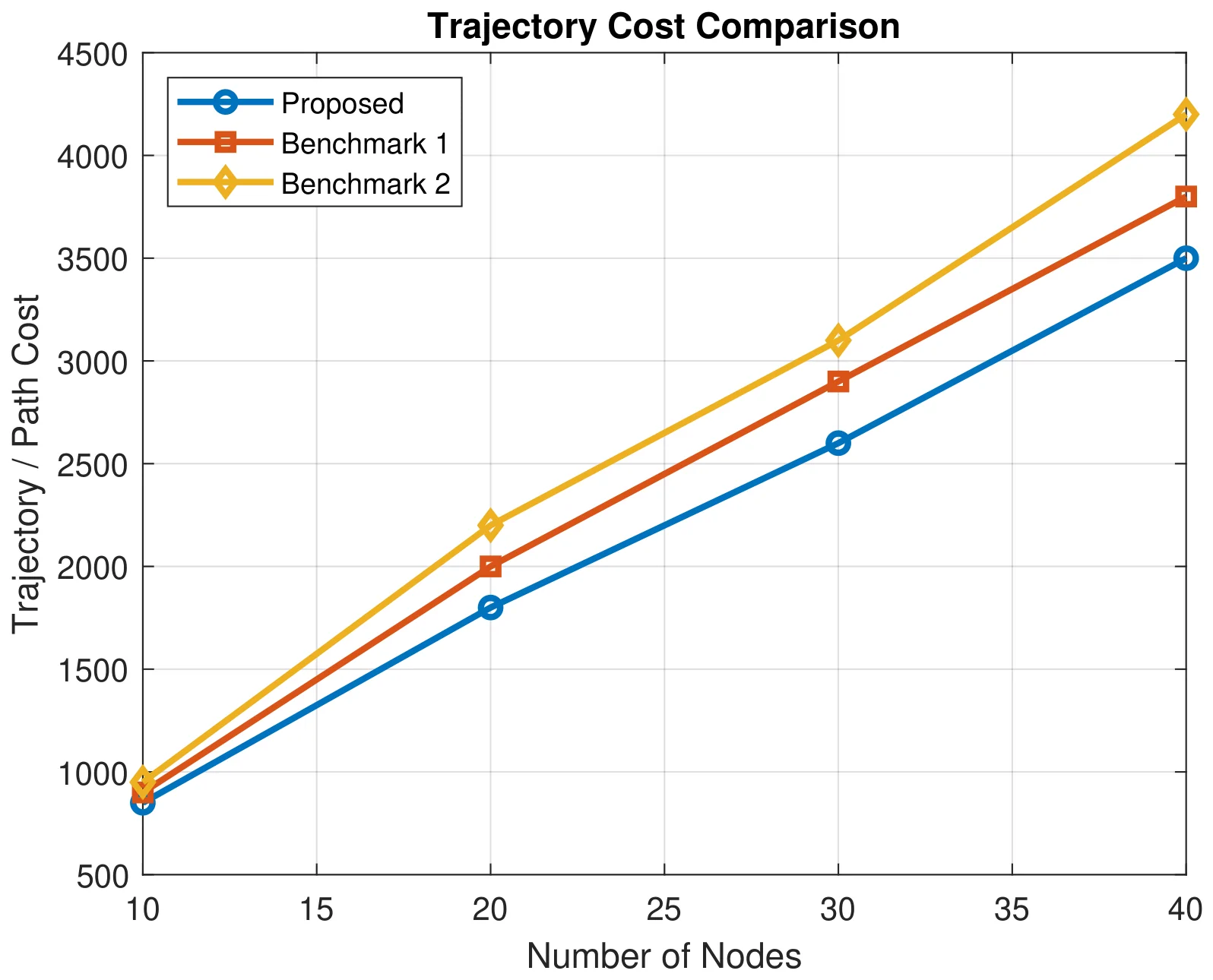
Trajectory and path-fitness comparison across different network sizes. The reported cost follows the GA fitness definition, which combines flight time, cumulative collection-time AoI, and trajectory-smoothness penalty under the shared benchmark environment.

The trajectory cost reported in Figure 9 follows the same GA path-fitness definition used for all compared methods. It is computed as a weighted combination of flight time, cumulative collection-time AoI, and trajectory-smoothness penalty:

(110) $$F_{\mathrm{path}}=w_{d}T_{\mathrm{fly}}+w_{a}\Delta_{\mathrm{AoI}}+w_{s}S_{\mathrm{smooth}},$$

where $w_{d}=1.0$, $w_{a}=0.5$, and $w_{s}=0.3$. Candidate trajectories that fail to cover the assigned sensing neighbourhoods are treated as infeasible and assigned $F_{\mathrm{path}}=\infty$. This definition ensures that the comparison reflects not only travelled distance, but also data freshness and route smoothness under the shared benchmark environment.

**Figure 10:**
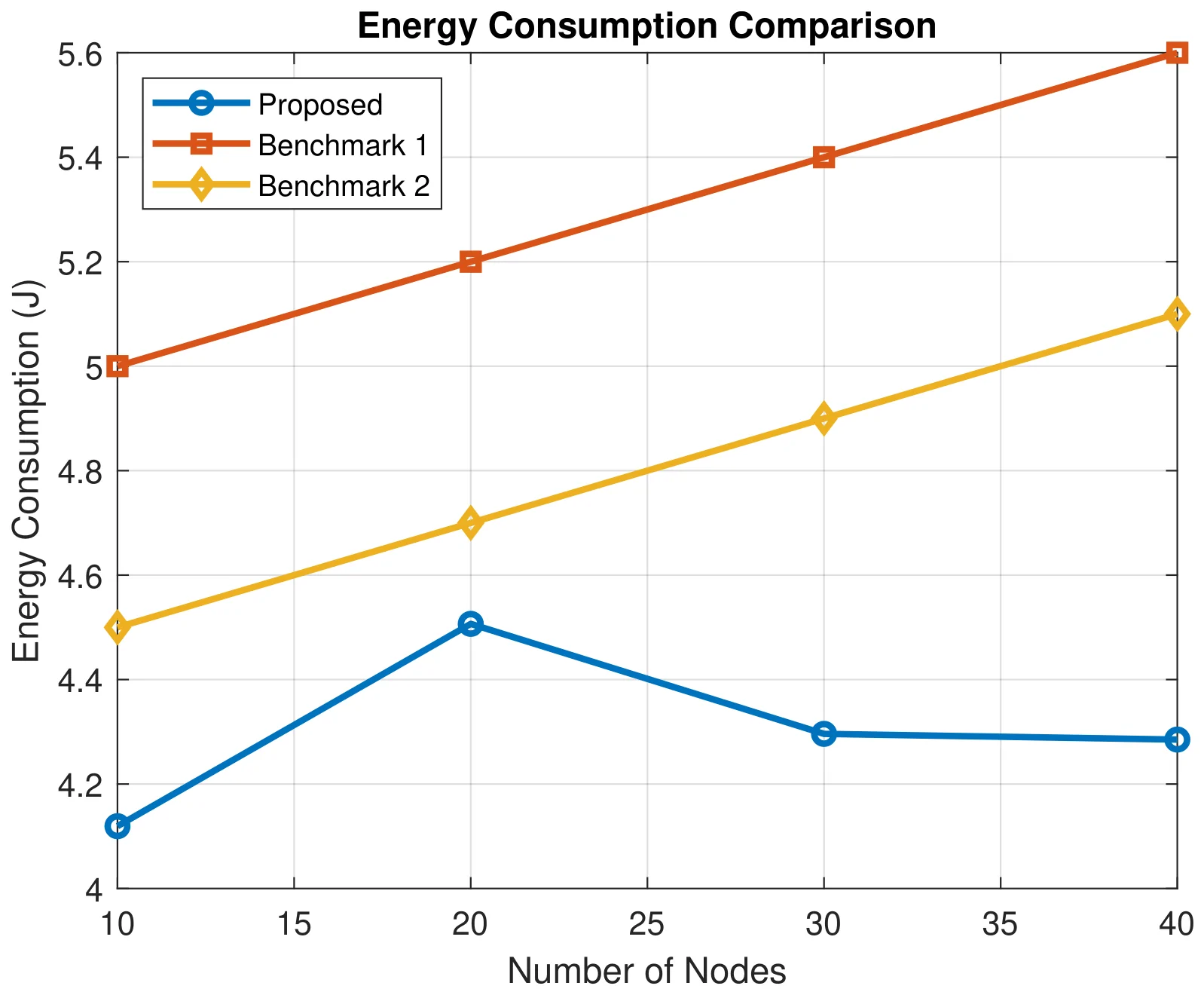
Average energy consumption per task across different network sizes for the proposed framework and the two benchmark methods.

Figure 10 compares the average energy consumption per task across different network sizes, while Figure 9 presents the corresponding trajectory and path costs. Under the evaluated conditions, both Figures demonstrate the consistent performance advantages of the proposed framework as the network size increases. As shown in Figure 10, the proposed framework achieves the lowest per-task energy consumption across all evaluated node configurations, indicating improved energy efficiency and scalability. Similarly, Figure 9 shows that the proposed framework maintains the lowest trajectory and path cost, suggesting that its spatial-planning strategy generates more efficient UAV routes than Benchmark 1 and Benchmark 2.

### 6.12. Overall Discussion

The experimental results show that the proposed framework provides a balanced and effective mission-level solution compared with both planning-oriented and MEC-oriented benchmark methods. Against the modified GA planning baseline, the proposed framework achieved the same 100% sensing success rate and collision-free execution, while reducing average AoI from 183.74 s to 83.93 s, total collection time from 396.29 s to 168.91 s, and total energy consumption from 265.91 J to 244.50 J. These results demonstrate that GA-based task allocation and route sequencing alone are not sufficient for time-critical disaster-response sensing, especially when information freshness and execution efficiency are considered.

Against the Benchmark 2-adapted cooperative MEC baseline, the proposed framework also maintained complete sensing coverage with no dropped tasks, while achieving lower AoI, lower delay, lower processing delay, lower energy consumption, and lower system cost. Specifically, the proposed framework reduced average AoI from 126.84 s to 83.93 s, average delay from 40.49 s to 8.45 s, average processing delay from 40.07 s to 7.46 s, total energy consumption from 2119.53 J to 244.50 J, and system cost from 1399.15 to 21.31. Although the Benchmark 2-adapted baseline achieved slightly higher fairness and shorter computational runtime, the proposed framework provided substantially safer multi-UAV execution, as detailed in Section 6.11.

The most important observation is that the proposed framework does not rely on a single dominant metric. Instead, it jointly considers sensing success, AoI, delay, processing delay, energy consumption, queue stability, and safety-aware execution. This balance is particularly important in disaster-response scenarios, where a method that is computationally efficient but unsafe, or fast but energy-intensive and stale in information delivery, is not operationally sufficient. The proposed framework, therefore, provides a more practical trade-off between information freshness, energy efficiency, computation control, and safe multi-UAV coordination.

Overall, the reported results support the central premise of this work: a layered spatial–temporal–safety decision framework is more suitable for realistic multi-UAV disaster monitoring than fragmented optimisation of route planning or computation management alone. By integrating 3D-TSPN sensing neighbourhoods, AoI-aware GA sequencing, RRT-Connect path feasibility checking, Lyapunov-based binary MEC offloading, queue-stability control, and multi-UAV safety monitoring, the proposed framework offers a more complete and operationally robust solution for disaster-response data collection in complex 3D environments.

Table 17 provides a qualitative comparison of the proposed framework with the two adapted benchmark methods. The comparison clarifies their respective optimisation scopes, supported capabilities, principal strengths, and limitations.

## 7. Conclusions and Future Work

This paper proposes a layered spatial–temporal optimisation framework for resilient multi-UAV disaster-response missions in complex three-dimensional and communication-constrained environments. The proposed architecture integrates 3D-TSPN sensing-neighbourhood coverage, AoI-aware GA-based route sequencing, RRT-Connect path feasibility checking, Lyapunov-based binary MEC offloading, and continuous-time multi-UAV safety monitoring within a coordinated execution framework. By jointly considering sensing coverage, obstacle-aware path feasibility, information freshness, computation delay, energy consumption, queue behaviour, and operational safety, the framework addresses key limitations of fragmented UAV planning and computation-control approaches.

The experimental evaluation showed that the proposed framework provides a balanced mission-level solution for UAV-assisted disaster data collection under the considered simulation conditions. The spatial layer improves coverage flexibility by allowing UAVs to collect data from sensing neighbourhoods rather than exact sensor coordinates, while the AoI-aware GA promotes freshness-aware route sequencing. RRT-Connect supports obstacle-aware three-dimensional path generation, and the Lyapunov-based temporal layer dynamically selects between local processing and full MEC offloading according to queue backlog, delay, energy consumption, completion-time AoI, wireless-link feasibility, and task deadlines. The safety layer supports concurrent multi-UAV operation through inflated-obstacle modelling, continuous closest-approach checking, temporal desynchronisation, and bounded conflict-resolution actions.

The processing-policy evaluation further showed that MEC offloading is most effective as a selective option rather than as a universally dominant processing mode. Under light workloads and favourable onboard-computation conditions, all-local processing can remain competitive in terms of delay and system cost. However, the paired 20-seed evaluation showed that selective MEC use reduced mean energy consumption relative to all-local processing while avoiding the completion-time AoI degradation associated with excessive all-MEC operation. The proposed controller, therefore, retains local processing for tasks that can be executed efficiently onboard and activates MEC only when the current channel, queue, delay, and energy conditions make offloading the lower-cost feasible action.

The Monte Carlo evaluation over 20 independent random seeds provided additional evidence of the robustness of the complete framework. Across the evaluated proposed-policy runs, all missions remained valid, achieved complete sensing coverage, processed all collected tasks, and passed the final continuous-time safety audit after conflict resolution and trajectory validation. The adaptive recovery mechanism was activated in only one run and successfully preserved sensing coverage, obstacle feasibility, and inter-UAV safety. These outcomes should be interpreted as evidence under the stated stochastic simulation conditions rather than as universal guarantees for all possible disaster environments.

Overall, the results support the value of separating the framework into spatial, temporal, and safety layers while allowing these layers to interact through shared mission states. This structure improves interpretability and preserves a clear functional role for each component: 3D-TSPN provides sensing-coverage flexibility, the AoI-aware GA determines freshness-aware route ordering, RRT-Connect verifies obstacle-aware path feasibility, Lyapunov control manages binary local/MEC processing decisions, and the safety layer monitors and mitigates multi-UAV conflict risks. The analytical queue-stability and cost-optimality results remain conditional on the boundedness, feasibility, and stationary-policy assumptions stated in the Lyapunov analysis, while the simulation results provide complementary finite-horizon evidence of mission-level behaviour.

### Limitations, Practical Implications and Future Work

The reported findings should be interpreted within the assumptions of the simulation model and the layered solution approach. The proposed GA–RRT–Lyapunov–safety decomposition does not solve the complete coupled mission problem using a single exact optimisation method. Consequently, no global-optimality guarantee is claimed for the complete mission solution. Instead, the framework produces feasible and mission-valid solutions through coordinated layer-specific optimisation, route validation, temporal processing control, and fleet-level safety auditing. Future work will investigate exact formulations for tractable subproblems, lower bounds, optimality-gap analysis against small-scale benchmark instances, and stronger performance bounds for the complete layered procedure.

The GA and RRT-Connect components are stochastic and parameter-dependent. Their computational cost and solution quality may vary with the GA population size, generation and time budgets, crossover and mutation settings, RRT step size, node and sampling limits, goal bias, obstacle geometry, computational budget, and random seed. Although every retained route is revalidated for sensing coverage, map boundaries, inflated-obstacle clearance, path continuity, and inter-UAV separation, these checks establish feasibility rather than global route optimality. Future work will, therefore, conduct systematic parameter-sensitivity analysis, adaptive parameter and computational budget selection, improved warm-start strategies, deterministic refinements, and broader comparisons across random seeds and environmental configurations.

The evaluation is simulation-based and has not yet been validated using physical UAV hardware. In addition, the current experiments consider four UAVs, 20 sensor-associated tasks, a finite set of obstacle environments, synthetic stochastic wireless and workload conditions, and 20 independent random seeds. Although the Monte Carlo analysis improves statistical reliability under these settings, it does not establish universal performance across arbitrary maps, fleet sizes, wireless regimes, workloads, dynamic obstacles, or hardware platforms. Future evaluation will, therefore, include larger UAV fleets, denser sensing deployments, more diverse maps and obstacle densities, broader wireless and workload conditions, larger random-seed sets, and field-scale disaster-response scenarios.

The current spatial and safety models assume that the relevant static obstacles are represented in the planning map before mission execution. The adaptive minimum-cost recovery mechanism operates during pre-mission planning and repairs routes that are infeasible with respect to the known obstacles, map boundaries, sensing-neighbourhood coverage, or path-validity constraints. During mission execution, the safety layer can apply bounded waiting, speed, or altitude adjustments to mitigate predicted inter-UAV separation conflicts, but the framework does not perform perception-triggered online trajectory replanning for previously unknown, newly appearing, or moving obstacles. Future work will incorporate onboard environmental perception, dynamic map updating, event-triggered receding-horizon or incremental replanning from the UAV’s current position, velocity, residual-energy, and queue states, and fleet-wide safety revalidation after every trajectory modification.

The temporal model assumes indivisible tasks, binary LOCAL/MEC processing, fixed onboard CPU capacity, one aggregate queue per UAV, and a single static MEC node. CPU-frequency scaling, partial task offloading, parallel local–edge execution, explicit task deferral, and multi-server load balancing are not included. The single MEC node may also represent a point of failure because the current model evaluates wireless-link and processing feasibility but does not explicitly model an independent server-availability state. When MEC processing is unavailable or infeasible, LOCAL processing is selected whenever it satisfies the deadline and residual-energy constraints; if neither mode is feasible, the task is dropped. Future work will jointly optimise task-partition ratios, UAV CPU frequency, energy consumption, processing delay, and queue state. It will also consider multiple MEC nodes, heterogeneous server capacities and queues, node association, load balancing, wireless handover, backhaul latency, congestion, failure-aware redundancy, and task deferral.

The Lyapunov stability and cost results are conditional infinite-horizon statements based on bounded arrivals and service, bounded instantaneous cost, and the existence of feasible stationary stabilising and cost-optimal policies. The finite-duration queue traces provide empirical evidence of bounded mission-level behaviour under the evaluated conditions, but they do not independently prove strong queue stability for arbitrary arrival and service regimes. Future work will examine time-varying and bursty workloads, wider arrival and service regions, MEC-server congestion, non-stationary wireless conditions, and empirical validation of the theoretical energy–delay–AoI and backlog trade-offs. Adaptive and learning-assisted decision mechanisms will also be investigated while retaining the interpretability and explicit feasibility checks of the current Lyapunov-based controller.

The propulsion, communication, and computation-energy models use simplified and normalised parameters to support internally consistent algorithmic comparisons. Accordingly, the reported energy values should not be interpreted as hardware-calibrated estimates for a specific commercial UAV. Future studies will incorporate measured propulsion, communication, computation, battery-degradation, and environmental-disturbance data and will evaluate the sensitivity of route and processing decisions to energy-model uncertainty.

The quadrotor equations provide a physical interpretation of the mission-level position-reference trajectories, but the simulation does not explicitly propagate motor speeds, rotor saturation, attitude transients, angular-rate limits, detailed aerodynamic effects, or a hardware-specific low-level controller. Future work will integrate actuator and flight-envelope constraints, a validated low-level flight controller, hardware-in-the-loop simulation, and real UAV experiments involving onboard perception, localisation, communications, computation hardware, and battery-state estimation.

From a practical perspective, the framework provides a mission-level mechanism for coordinating sensing coverage, information freshness, computation control, queue behaviour, and multi-UAV safety. Its modular spatial–temporal–safety structure may allow individual planning, processing, or safety modules to be updated without redesigning the complete framework. Nevertheless, practical disaster-response deployment would additionally require reliable onboard perception and localisation, continuous map maintenance, communication-outage management, robust battery-state estimation, MEC-server availability monitoring, embedded computational feasibility, secure communication, regulatory compliance, and integration with certified low-level flight-control systems. The present results, therefore, demonstrate the value of the proposed layered integration under the stated simulation conditions rather than unrestricted operational readiness for all disaster-response scenarios.

Overall, these matched future directions preserve the modular spatial–temporal–safety architecture while extending its scalability, adaptability, physical fidelity, theoretical characterisation, and readiness for practical deployment.

## Figures and Tables

**Table 1:** Critical comparison of representative studies related to the proposed framework.

Study	Problem, Objective, and Method	Main Strength	Limitation Relative to This Work
Tran et al. [15]	Robust quadrotor path generation and tracking under uncertainties and disturbances using RRT*, integrated sliding-mode control, RBF neural compensation, and PSO tuning.	Combines collision-free reference-path generation with robust low-level tracking.	Does not address sensor assignment, AoI-aware sequencing, MEC offloading, queue control, or multi-UAV mission coordination.
Shi et al. [16]	Real-time UAV trajectory planning and tracking in static and dynamic multi-obstacle environments using Soft Actor–Critic to adaptively tune ANMPC cost weights and receding-horizon constrained control.	Provides adaptive closed-loop tracking, dynamic-obstacle avoidance, wind-disturbance robustness, and real-time control.	Focuses mainly on single-UAV local planning and tracking rather than multi-UAV sensor assignment, AoI, MEC offloading, queue control, and fleet-level safety coordination.
Baek et al. [21]	Maximises wireless-sensor-network lifetime by jointly optimising UAV hovering locations and durations through Lagrange-based optimisation and iterative route refinement.	Couples data collection, wireless charging, UAV energy, and sensor residual energy.	Assumes one UAV, fixed altitude, and a predetermined visit order; AoI, MEC, 3D obstacles, and fleet safety are not considered.
Zhan and Zeng; Luo et al. [23,25]	Minimise multi-UAV data-collection completion time using min–max m-TSP, convex optimisation, time discretisation, SCA, and approximation algorithms.	Provides rigorous multi-UAV trajectory, hovering, travelling, scheduling, and sensor-association optimisation.	Does not jointly model AoI, computation queues, LOCAL/MEC processing, RRT-based obstacle avoidance, and continuous-time deconfliction.
Jia et al.; Abd-Elmagid et al.; Eldeeb et al. [22,27,28]	Minimise AoI through UAV route planning, update scheduling, and data-acquisition decisions using dynamic programming, MDP formulations, and deep reinforcement learning.	Directly incorporates information freshness into trajectory and scheduling decisions.	Uses simplified or discretised mobility and does not jointly include obstacle-aware 3D paths, MEC processing, queues, and fleet-level safety.
Ning et al. [31]	Minimises system-wide computation cost by jointly optimising multi-user offloading and UAV–MEC server deployment through stochastic games and distributed learning.	Captures the coupling between task offloading and multiple mobile MEC-server locations.	Treats UAVs mainly as MEC servers and does not optimise sensing-neighbourhood visits, AoI-aware routes, or obstacle-aware mission trajectories.
Xu et al. [32]	Jointly optimises multi-UAV trajectories and cooperative computation resources using PPO, greedy association, and SCA under moving users and random task arrivals.	Integrates cooperative UAV computation, trajectory planning, delay, energy, association, and resource allocation.	Does not consider sensing-neighbourhood coverage, AoI-aware sensor order, RRT-based obstacle avoidance, or continuous-time safety auditing.
Carrese et al. [36]	Coordinate UAV seeking and human beautification actions for improperly parked e-scooters while maximising total operational value using a time–space MILP with unsplittable multicommodity flow, movement-arc reduction, and RINS-based warm-start improvement.	Provides a rigorous joint scheduling model with UAV batteries, battery swapping, human–UAV interactions, and exact-solver support.	Targets e-scooter fleet beautification rather than disaster sensing and does not model AoI, MEC processing, computation queues, obstacle-aware 3D routes, or continuous fleet safety.
Phillips and Likhachev; Stern et al. [34,35]	Address collision-free dynamic and multi-agent path planning using safe intervals and graph-based MAPF formulations.	Provide systematic conflict modelling and theoretical completeness or optimality under their assumptions.	Rely mainly on discretised graphs or configurations and do not optimise sensing coverage, AoI, energy, MEC decisions, or computation queues.
Proposed framework	Integrates 3D-TSPN, AoI-aware GA sequencing, RRT-Connect, Lyapunov binary LOCAL/MEC control, adaptive route recovery, and continuous-time safety auditing.	Connects sensing, spatial planning, AoI, computation, queues, energy, and multi-UAV safety through shared mission states.	Uses hierarchical heuristics without a global-optimum guarantee and currently assumes known static obstacles, indivisible tasks, fixed CPU capacity, and one MEC node.

**Table 2:** Simulation Parameters and Default Values.

Parameter	Value
Disaster region size	$100\times 100\times 60\;m^{3}$
Number of sensors *N*	20
Number of UAVs *U*	4
Sensor sensing radius $R_{s}$	8m
Sensor service duration	0 s
UAV speed $v_{\mathrm{uav}}$	1m/s
Battery capacity $E_{\max}$	1000J
Safety margin $d_{\mathrm{safe}}$	4m
Minimum UAV separation	10m
Collision avoidance radius	20m
Slowdown factor	0.7
Spatial deconfliction altitude step	8 m
Maximum spatial deconfliction altitude shift	20 m
Number of obstacle towers	3
MEC node position	(50,50,5)
Data size per sensor	8Mbits
Carrier frequency	2.4GHz
Dynamic bandwidth range	0.5–3.0MHz
Bandwidth jitter	0.30
Link outage probability	0.20
Path-loss exponent $\eta_{\mathrm{pl}}$	2.5
Minimum transmit power	0.1W
Maximum transmit power	1.0W
UAV CPU capacity	2.0GHz
MEC CPU capacity	5.0GHz
CPU cycles per bit	2000cycles/bit
Maximum task deadline $\tau_{\max}$	20s
GA population size	60
GA maximum generations	120
GA time limit per UAV	25s
GA crossover probability	0.85
Base mutation rate	0.20
Elite rate	0.15
RRT step size	5m
RRT maximum nodes	400
RRT goal bias probability	0.20
Lyapunov control parameter *V*	8.0
UAV flight or hovering power $P_{i}^{\mathrm{fly}}$	0.2W
UAV computation power $P_{i}^{\mathrm{cpu}}$	0.6W
Reference channel gain $h_{0}$	$5\times 10^{-7}$
Noise power spectral density $N_{0}$	$1\times 10^{-21}\;W/\mathrm{Hz}$
Instantaneous noise power $\sigma_{i}^{2}\left(t\right)$	$5\times 10^{-16}$–$3\times 10^{-15}\;W$
Instantaneous noise power in dBm	−123.01 to −115.23dBm
Base interference power $I_{\mathrm{base}}$	$1.5\times 10^{-11}\;W$
Interference burst probability	0.40
Interference burst multiplier	30
Simulation time-step duration Δt	1.0s
GA weights $w_{d},w_{a},w_{s}$	1.0,0.5,0.3
GA feasibility handling	Hard rejection, F=∞
Lyapunov weights α,β,γ	0.4,0.4,0.2
Default illustrative simulation seed	42
Clustering initialisation	Fixed clustering seed 0 for all scenarios and all Monte Carlo seeds
Number of independent Monte Carlo seeds	20
Processing-control scenarios per seed	PROPOSED, ALL_LOCAL, and ALL_MEC
Total number of scenario executions	20×3=60
Random-seed approach	Twenty independent simulation seeds were used. Within each seed, the same stochastic realisation was reused across PROPOSED, ALL_LOCAL, and ALL_MEC to enable paired comparisons under identical parameters and computational budgets.
UAV start-time offsets	[0,25,50,75]s

**Table 3:** Comparative Performance Across Planning Configurations.

Method	UAVs	Success	Avg. AoI (s)	Energy (J)	Mission Completion Time (s)	Collisions
Proposed Full Framework	4	100.0%	74.24	266.96	210.66	0
Nearest Neighbour Baseline	4	85.0%	145.76	325.81	293.80	17
Single-UAV Baseline	1	100.0%	151.56	189.09	460.92	0

**Table 4:** Performance comparison between single-UAV and four-UAV configurations of the proposed framework.

Metric	Single	4 UAVs	Change
Success rate	100.0%	100.0%	No change
Sensors collected	20	20	No change
Average AoI (s)	151.56	74.24	51.0% red.
Energy (J)	189.09	266.96	41.2% inc.
Mission completion time (s)	460.92	210.66	54.3% red.
Obstacle collisions	0	0	No change
Inter-UAV collisions	N/A	0	Collision-free

**Table 5:** Binary Processing Decisions and Final Queue Backlogs.

Metric	Value
LOCAL decisions	19
MEC-offloading decisions	1
Dropped tasks	0
Final queue backlog, UAV 1	4.50
Final queue backlog, UAV 2	5.33
Final queue backlog, UAV 3	4.49
Final queue backlog, UAV 4	7.12

**Table 6:** Comparison of the proposed framework with the nearest-neighbour and single-UAV baselines.

Metric	Compared with Nearest Neighbour	Compared with Single UAV
Success rate	+15 percentage points	No change
Average AoI	49.1% reduction	51.0% reduction
Energy consumption	18.1% reduction	41.2% increase
Collection time	28.3% reduction	54.3% reduction
Obstacle collisions	Reduced from 17 to 0	No change
Dropped tasks	Reduced from 3 to 0	No change

**Table 7:** Safety and collision-monitoring results of the proposed multi-UAV framework.

Safety Metric	Value
Real obstacle collisions	0
Inter-UAV collisions	0
Near misses	0
Buffer-zone intrusions	0

**Table 8:** Controlled single-seed safety ablation separating the effects of fixed UAV start-time offsets and active conflict resolution.

Configuration	Initial Unsafe	Final Collisions	Final Near Misses	Min. Separation (m)	Mission Completion Time (s)	Resolution Actions	Valid
No offsets, no resolution	1	1	0	1.095	207.01	0	No
Fixed offsets only	0	0	0	11.015	268.62	0	Yes
Resolution only	1	0	0	10.079	214.12	1	Yes
Offsets + resolution	0	0	0	11.015	268.62	0	Yes

**Table 9:** System-level robustness metrics of the proposed multi-UAV framework.

Metric	Observed Value
Coverage success rate	100.0%
Average AoI	74.24 s
Total collection time	210.66 s
Total energy consumption	266.96 J
Local-processing decisions	19
MEC-offloading decisions	1
Dropped tasks	0
Final queue backlog	4.49–7.12 tasks
Real obstacle collisions	0
Inter-UAV collisions	0
Near misses	0
Buffer-zone intrusions	0

**Table 10:** Monte Carlo robustness results over 20 independent random seeds.

Metric	Mean	Standard Deviation	95% CI
Completion-time AoI (s)	142.694	17.740	[134.391, 150.997]
Mission completion time (s)	314.098	39.875	[295.436, 332.760]
Total energy consumption (J)	257.204	10.618	[252.235, 262.174]

**Table 11:** Mission-validity and safety outcomes across the 20-seed evaluation.

Outcome	Observed Value
Successfully completed runs	20/20
Mission-validity rate	100%
Sensor coverage rate	100%
Runs with dropped tasks	0
Final collision events	0
Final near-miss events	0
Runs passing the all-pair safety audit	20/20
Minimum observed inter-UAV separation	10.002 m
Required safety threshold	10 m
Recovery activations	1/20

**Table 12:** Paired processing-policy comparison across 20 independent random seeds. Values are reported as mean ± standard deviation.

Metric	Proposed	All-local	All-MEC
Completion-time AoI (s)	142.694±17.740	142.787±17.723	145.880±18.147
Mission completion time (s)	314.098±39.875	314.098±39.875	314.098±39.875
Total energy consumption (J)	257.204±10.618	262.121±10.760	249.524±22.289
MEC-use rate (%)	5.25±4.99	0.00±0.00	54.75±8.96
Mission-valid runs	20/20	20/20	20/20
Runs with complete coverage	20/20	20/20	20/20
Runs with dropped tasks	0/20	0/20	0/20

**Table 13:** Parameter settings used in the three MEC-offloading sensitivity regimes.

Parameter	Local-Processing-Favourable	Balanced	MEC-Favourable
UAV CPU capacity (GHz)	3.5	1.8	0.8
MEC CPU capacity (GHz)	4.0	7.0	12.0
Task size (Mbit)	6	8	10
CPU cycles per bit	1600	2000	2600
Local-processing power (W)	0.35	0.85	1.40
Bandwidth range (MHz)	0.4–1.5	1–5	4–12
Link-outage probability	0.35	0.10	0.02
Initial queue backlog	0	2	5
Bandwidth-boost probability	0.05	0.25	0.50
Maximum processing deadline (s)	20	22	30

**Table 14:** MEC-offloading sensitivity results across three operating conditions using the same 20 matched random seeds. Values are reported as mean ± standard deviation.

Metric	Local-Processing- Favourable	Balanced Local/MEC	MEC- Favourable
LOCAL rate (%)	100.00±0.00	46.00±11.99	0.25±1.12
MEC rate (%)	0.00±0.00	54.00±11.99	98.00±3.40
Processing delay (s)	2.716±0.084	6.786±0.531	2.424±0.255
Completion-time AoI (s)	137.58±17.79	141.65±17.78	134.67±19.13
Total energy consumption (J)	186.06±10.80	238.21±21.43	169.36±12.51
Final queue backlog	1.108±0.168	1.432±0.277	1.096±0.139
Post-collection dropped tasks per run	0.00±0.00	0.00±0.00	0.35±0.67

Note: The three operating conditions were evaluated using the same 20 matched random seeds. In the MEC-favourable condition, the LOCAL and MEC rates do not sum to 100% because a small proportion of the collected tasks were infeasible under both processing modes and were, therefore, dropped during post-collection processing. This does not indicate a loss of sensing coverage, since all sensor updates were successfully collected.

**Table 15:** Comparison with the Modified GA Planning Baseline Inspired by Benchmark 1.

Metric	Proposed Framework	Modified GA Baseline	Better Result
Success rate	100.0%	100.0%	Equal
Sensors collected	20/20	20/20	Equal
Average AoI (s)	83.93	183.74	Proposed
Mission completion time (s)	168.91	396.29	Proposed
Total energy consumption (J)	244.50	265.91	Proposed
Average energy per collected sensor (J)	12.23	13.30	Proposed
Obstacle collisions	0	0	Equal
Inter-UAV collisions	0	0	Equal
Buffer-zone intrusions	0	0	Equal
Dropped tasks	0	0	Equal
Fairness index	0.9705	0.9804	Modified GA

**Table 16:** Performance comparison between the proposed framework and the Benchmark 2-adapted cooperative MEC baseline.

Metric	Proposed Framework	Benchmark 2-Adapted SCA/CVX	Better Result
Success rate	100.0%	100.0%	Equal
Sensors collected	20/20	20/20	Equal
Mission completion time (s)	168.91	191.05	Proposed
Average AoI (s)	83.93	126.84	Proposed
Average delay per sensor (s)	8.45	40.49	Proposed
Average processing delay (s)	7.46	40.07	Proposed
Total energy consumption (J)	244.50	2119.53	Proposed
Average energy per task (J)	12.23	105.98	Proposed
System cost	21.31	1399.15	Proposed
Jain fairness index	0.9705	1.0000	Benchmark 2
Weighted fairness index	0.9613	0.9997	Benchmark 2
Obstacle collisions	0	0	Equal
Inter-UAV collisions	0	24	Proposed
Near misses	2	7	Proposed
Buffer-zone intrusions	0	0	Equal
Dropped tasks	0	0	Equal
Runtime (s)	37.53	1.53	Benchmark 2
CVX solver used	N/A	Yes	N/A

**Table 17:** Qualitative comparison of the proposed framework with the two adapted benchmark methods.

Metric	Proposed Framework	Benchmark 1: Modified GA Planning	Benchmark 2: Adapted Cooperative MEC Optimisation
Core optimisation scope	3D-TSPN sensing coverage, AoI-aware GA sequencing, RRT-Connect path feasibility, Lyapunov-based binary MEC offloading, and safety monitoring.	modified GA-based integrated multi-UAV task allocation and path planning with resource-related and simultaneous-arrival constraints.	Benchmark 2-inspired cooperative MEC optimisation using an adapted long-timescale UAV-positioning stage, greedy association, SCA/CVX-based resource allocation, and cooperative task offloading.
Sensing coverage	Explicitly modelled using 3D sensing neighbourhoods around sensors.	Addressed indirectly as task/target assignment and visitation, but not modelled as sensing-neighbourhood coverage.	Not the primary focus.
Obstacle-aware 3D feasibility	Explicitly addressed using RRT-Connect and inflated obstacle models.	Limited in the disaster-response sense; the benchmark focuses on flyable planning and task execution rather than 3D-obstacle-rich sensing environments.	Not the primary focus.
AoI awareness	Explicitly included in route sequencing and task completion evaluation.	Not explicitly addressed.	Not explicitly addressed.
Computation offloading	Binary local/MEC decision using Lyapunov-based temporal control.	Not addressed; the benchmark is planning-oriented.	Central component, including cooperative computation and inter-UAV task offloading.
Queue stability	Explicitly addressed through Lyapunov drift-plus-penalty control.	Not addressed.	Not explicitly addressed as Lyapunov queue stability; computation delay and resource allocation are optimised instead.
Mission/ collection time	Reported from the proposed simulation output.	Reported or represented through planning-efficiency measures such as mission completion time and route cost.	Not reported as direct sensing mission collection time; the focus is computation-task completion and system cost.
Average AoI	Reported from the proposed simulation output.	Not reported.	Not reported.
Average delay	Reported under spatial execution and binary offloading constraints.	Not reported in the same computation-processing form.	Reported as a computation-performance metric.
Energy consumption	Includes flight, local-processing, and MEC-transmission energy.	Represented mainly through distance- or fuel-related planning constraints rather than detailed computation or communication energy.	Central to the energy–delay trade-off analysis.
Fairness	Evaluated under combined sensing, trajectory, and computation constraints.	Not a primary metric, although the maximum route-length term can indirectly reduce workload imbalance.	Strongly emphasised under cooperative MEC and load-balancing settings.
Scalability	Evaluated by increasing sensing demand, UAV workload, and environmental complexity.	Evaluated mainly through planning complexity, number of UAVs/targets, and mission-completion behaviour.	Evaluated mainly through network size, task demand, available resources, and environmental dynamics.
Safety	Includes obstacle-collision checking, inter-UAV monitoring, and buffer-zone analysis.	Limited or not reported in the same continuous 3D safety-monitoring form.	Not usually a primary metric.
Main strength	Balanced mission-level optimisation across sensing coverage, information freshness, computation control, and safety.	Strong integrated task-allocation and route-planning capability using a modified GA.	Strong cooperative computation, trajectory planning, and resource allocation under MEC constraints.
Main limitation	Requires more comprehensive evaluation because spatial planning, temporal control, and safety monitoring interact within one framework.	Limited integration of AoI, MEC processing, queue stability, and continuous 3D safety monitoring.	Limited integration of obstacle-aware 3D route realisation, sensing-neighbourhood coverage, and information freshness.

## Data Availability

The data supporting the findings of this study are available from the corresponding author upon reasonable request.

## Appendix A. Proof of the Lyapunov Performance Result

This appendix provides the complete proof of Theorem 1.

### Appendix A.1. One-Step Drift Bound

From the queue evolution,

(A1) $$Q_{i}\left(t+1\right)=\max\left[Q_{i}\left(t\right)-\mu_{i}\left(t\right),0\right]+A_{i}\left(t\right),$$

we have

(A2) $$\begin{array}{cc} Q_{i}^{2}\left(t+1\right) & ={\left(\max\left[Q_{i}\left(t\right)-\mu_{i}\left(t\right),0\right]+A_{i}\left(t\right)\right)}^{2}\\ & \leq {\left(Q_{i}\left(t\right)-\mu_{i}\left(t\right)\right)}^{2}+A_{i}^{2}\left(t\right)+2A_{i}\left(t\right)Q_{i}\left(t\right)\\ & =Q_{i}^{2}\left(t\right)+\mu_{i}^{2}\left(t\right)+A_{i}^{2}\left(t\right)+2Q_{i}\left(t\right)\left(A_{i}\left(t\right)-\mu_{i}\left(t\right)\right). \end{array}$$

The inequality follows because $\max\left[Q_{i}\left(t\right)-\mu_{i}\left(t\right),0\right]\leq Q_{i}\left(t\right)$ and ${\left(\max[x,0]\right)}^{2}\leq x^{2}$.

Subtracting $Q_{i}^{2}\left(t\right)$, summing over *i*, multiplying by 1/2, and conditioning on Q(t) yields

(A3) $$\begin{array}{cc} \Delta (Q(t)) & \leq \frac{1}{2}\sum_{i=1}^{U}E\left[A_{i}^{2}\left(t\right)+\mu_{i}^{2}\left(t\right)|Q\left(t\right)\right]\\ & +\sum_{i=1}^{U}Q_{i}\left(t\right)E\left[A_{i}\left(t\right)-\mu_{i}\left(t\right)|Q\left(t\right)\right]. \end{array}$$

Using the deterministic bounds in Assumption 1 gives

(A4) $$\Delta \left(Q\left(t\right)\right)\leq B+\sum_{i=1}^{U}Q_{i}\left(t\right)E\left[A_{i}\left(t\right)-\mu_{i}\left(t\right)|Q\left(t\right)\right],$$

where

(A5) $$B=\frac{1}{2}\sum_{i=1}^{U}\left[{\left(A_{i}^{\max}\right)}^{2}+{\left(\mu_{i}^{\max}\right)}^{2}\right].$$

Adding the conditional weighted penalty gives

(A6) $$\begin{array}{cc} \; & \Delta \left(Q\left(t\right)\right)+VE\left[g(t)|Q(t)\right]\\ \; & \leq B+\sum_{i=1}^{U}Q_{i}\left(t\right)E\left[A_{i}\left(t\right)-\mu_{i}\left(t\right)|Q\left(t\right)\right]+VE\left[g(t)|Q(t)\right]. \end{array}$$

### Appendix A.2. Proof of the Cost Bound

Because the analytical drift-plus-penalty policy considered in Theorem 1 minimises the decision-dependent right-hand side of (A6), its value cannot exceed the value obtained by substituting any other feasible policy. Substituting the stationary cost-optimal policy $\pi^{★}$ and using

(A7) $$E\left[A_{i}\left(t\right)-\mu_{i}^{\pi^{★}}\left(t\right)\right]\leq 0$$

gives

(A8) $$\Delta \left(Q\left(t\right)\right)+VE\left[g\left(t\right)\right]\leq B+Vg^{★}.$$

Taking full expectation and summing over t=0,…,T−1 yields

(A9) $$\begin{array}{cc} \; & E\left[L\left(Q\left(T\right)\right)\right]-E\left[L\left(Q\left(0\right)\right)\right]+V\sum_{t=0}^{T-1}E\left[g\left(t\right)\right]\\ \; & \leq TB+TVg^{★}. \end{array}$$

Since L(Q(T))≥0,

(A10) $$\frac{1}{T}\sum_{t=0}^{T-1}E\left[g\left(t\right)\right]\leq g^{★}+\frac{B}{V}+\frac{E[L(Q(0))]}{VT}.$$

Taking ${lim sup}_{T\to \infty }$ gives

(A11) $${\bar{g}}^{\mathrm{LBO}}\leq g^{★}+\frac{B}{V}.$$

### Appendix A.3. Proof of Strong Queue Stability

To establish a backlog bound, compare the proposed controller with the stationary ϵ-slack policy $\pi^{\epsilon }$. Under Assumption 3,

(A12) $$E\left[A_{i}\left(t\right)-\mu_{i}^{\pi^{\epsilon }}\left(t\right)\right]\leq -\epsilon .$$

Substitution into (A6) gives

(A13) $$\Delta \left(Q\left(t\right)\right)+VE\left[g\left(t\right)\right]\leq B-\epsilon \sum_{i=1}^{U}Q_{i}\left(t\right)+Vg^{\epsilon }.$$

Using the lower bound $E\left[g\left(t\right)\right]\geq g_{\min}$ gives

(A14) $$\Delta \left(Q\left(t\right)\right)\leq B+V\left(g^{\epsilon }-g_{\min}\right)-\epsilon \sum_{i=1}^{U}Q_{i}\left(t\right).$$

Taking expectation, summing from t=0 to T−1, and telescoping the Lyapunov terms yields

(A15) $$\begin{array}{cc} \epsilon \sum_{t=0}^{T-1}\sum_{i=1}^{U}E\left[Q_{i}\left(t\right)\right] & \leq T\left[B+V(g^{\epsilon }-g_{\min})\right]\\ & \;+E[L(Q(0))]. \end{array}$$

Dividing by ϵT gives

(A16) $$\begin{array}{cc} \frac{1}{T}\sum_{t=0}^{T-1}\sum_{i=1}^{U}E\left[Q_{i}\left(t\right)\right] & \leq \frac{B+V(g^{\epsilon }-g_{\min})}{\epsilon }\\ & +\frac{E[L(Q(0))]}{\epsilon T}. \end{array}$$

Taking ${lim sup}_{T\to \infty }$ establishes

(A17) $$\underset{T\to \infty }{lim sup}\frac{1}{T}\sum_{t=0}^{T-1}\sum_{i=1}^{U}E\left[Q_{i}\left(t\right)\right]\leq \frac{B+V(g^{\epsilon }-g_{\min})}{\epsilon }<\infty .$$

Therefore, the proposed controller provides the standard conditional strong-stability result under the stated boundedness, feasibility, and stationary-policy assumptions.
